# Phosphorylation of FBXL3 mediates GLDC polyubiquitination to suppress MHC-I expression and promote cancer immune evasion

**DOI:** 10.1016/j.cellin.2026.100308

**Published:** 2026-02-03

**Authors:** Rui Liu, Shu Li

**Affiliations:** Department of Infectious Diseases, Medical Research Institute, Zhongnan Hospital of Wuhan University, Frontier Science Center for Immunology and Metabolism, Taikang Center for Life and Medical Sciences, Wuhan University, Wuhan 430071, Hubei, China

**Keywords:** MHC-I antigen presentation, Immune escape, GLDC, FBXL3, ICB therapy

## Abstract

Glycine decarboxylase (GLDC) is overexpressed in multiple tumor types and contributes to tumorigenesis or immune evasion by unclarified mechanisms. Here we report that GLDC is polyubiquitinated at K636 following EGFR activation, which drives GLDC-dependent transcriptional inhibition of MHC-I genes and induces tumor cells to evade CD8^+^ T cell-mediated immunosurveillance. Mechanistically, EGFR activation triggers SRC-mediated FBXL3 phosphorylation at Y306, enabling its interaction with GLDC in nucleus. FBXL3 targets GLDC^K636^ for K63-linked polyubiquitination, and promotes the interaction of GLDC with SMARCE1/DMAP1 to inhibit STAT1-triggered transcriptional activation, resulting in transcriptional inhibition of downstream MHC-I genes. Phosphorylation of FBXL3^Y306^ decreases MHC-I levels in tumor cells and inhibits CD8^+^ T cells immunity in tumors. Consistently, inhibitors of SRC improves tumor-specific CD8^+^ T cells functions in TME and sensitizes antitumor effects of anti-PD-1 therapy. Our findings reveal an SRC-FBXL3-GLDC-MHC-I regulatory circuit that underlies CD8^+^ T cells immune evasion, and provide a potential therapeutic target to enhance ICB therapy.

## Highlights

• GLDC is polyubiquitinated at K636 following EGFR activation.
• EGFR activation triggers SRC-mediated FBXL3 phosphorylation at Y306.
• Phosphorylation of FBXL3^Y306^ targets GLDC^K636^ for K63-linked polyubiquitination.
• GLDC^K636^ polyubiquitination and FBXL3^Y306^ phosphorylation decrease MHC-I levels and inhibit CD8^+^ T cells immunity in tumors.

## Graphical abstract

**Figure:**
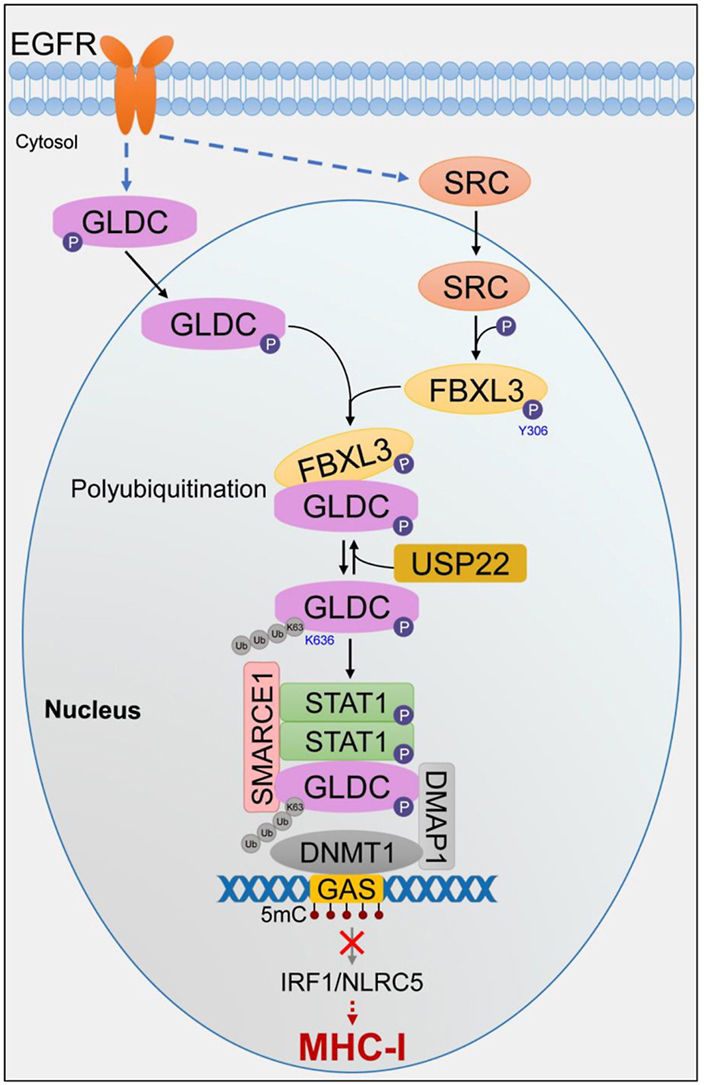

## 1 Introduction

Ubiquitination is a critical posttranslational modification that regulates diverse protein functions, such as protein stability and activity (Akutsu et al., 2016; Chen et al., 2019). This process involves a sequential enzymatic cascade consisting of E1 ubiquitin-activating enzyme, E2 ubiquitin-conjugating enzyme, and E3 ubiquitin ligase (Dikic et al., 2009). The removal of ubiquitin from the substrate is mediated by a class of deubiquitinating enzymes (DUBs) (Mevissen and Komander, 2017). The ubiquitin system governs various fundamental biological processes, such as signal transduction, gene transcription, and cell cycle progression (Akutsu et al., 2016). Deregulation of the ubiquitin system leads to the development of multiple human diseases, including cancer (Zheng and Shabek, 2017). The Skp1-Cul1-F-box protein (SCF) complex is one of the most well characterized types of E3 ubiquitin ligase complexes, whose activity is regulated by complex assembly (Zhou et al., 2013). It consists of four subunits: Skp1, Cul1, Roc1 and an F-box protein. The F-box protein serves as the substrate recognition component and each F-box protein recognizes distinct substrate groups. F-Box and Leucine Rich Repeat Protein 3 (FBXL3) is a part of the SCF ubiquitin ligase complex, which is thought to be responsible for the ubiquitylation and consequent degradation of cryptochromes (CRY1 and CRY2) and thus regulates oscillation of the circadian clock (Chen et al., 2021; Parico and Partch, 2020; Xing et al., 2013). Its deficiency causes circadian phenotypes in mice and behavioral problems (Shi et al., 2013). Beyond its circadian function, FBXL3 has been reported to play an oncogenic role in various human cancers, including liver, breast, prostate, lung, and colorectal cancers and glioblastoma (Chen et al., 2021; Kar et al., 2021). The level of FBXL3 in tumor cells is often inversely correlated with tumor growth (Guo et al., 2017; Huber et al., 2016; Wang et al., 2019). While the functions of FBXL3 in circadian regulation and tumor growth have been intensively studied, its potential roles in other important cellular processes, such as cancer immune evasion, remains unexplored.

Glycine decarboxylase (GLDC) is a key enzyme of glycine cleavage system that converts glycine into one-carbon units (Hiraga and Kikuchi, 1980; Liu et al., 2021). Mutations in GLDC gene cause glycine accumulation, leading to neural tube defect and glycine encephalopathy (also known as nonketotic hyperglycinemia) (Hellani et al., 2008). In addition, GLDC is frequently overexpressed in various human cancers and plays important roles in tumor growth. For example, high levels of GLDC in human non-small cell lung cancer (NSCLC) mediate tumorigenesis by promoting glycolysis, pyrimidine biosynthesis and sarcosine production (Zhang et al., 2012). In hepatocellular carcinoma, GLDC promotes tumor growth by sustaining protein lipoylation and mitochondrial activity (Zhang et al., 2012). In MYCN-amplified neuroblastomas, GLDC is significantly overexpressed, and is essential for tumor cell proliferation and tumorigenicity (Alptekin et al., 2019). In glioblastoma, GLDC activity is regulated by coordinated posttranslational modifications including acetylation and polyubiquitination, which contributes to regulation of glycine metabolism and glioma tumorigenesis (Liu et al., 2021).

Besides its roles in tumor progression, GLDC also acts as a transcriptional repressor to facilitate cancer immune evasion. Epidermal growth factor receptor (EGFR) activation triggers SRC-mediated phosphorylation and nuclear translocation of GLDC. The nuclear GLDC hijacks the STAT1 co-activator SMARCE1 to inhibit the binding of STAT1 to promoter regions of *IRF1* and *NLRC5* genes, and the GLDC/SMARCE1/STAT1 complex also recruits DMAP1/DNMT1 to induce promoter DNA hypermethylation of *IRF1* and *NLRC5* genes, resulting in transcriptional inhibition of the downstream MHC-I genes. Inhibition of GLDC restores MHC-I expression and inhibits immune evasion (Liu et al., 2025). While these findings clearly demonstrated the function of nuclear GLDC in regulating transcription of MHC-I genes, the post-translational mechanisms controlling its nuclear activity is unknown.

In this study, we found that nuclear GLDC binds to SRC-phosphorylated FBXL3 upon EGFR activation, leading to FBXL3-dependent polyubiquitination of GLDC, which contributes to GLDC-mediated transcriptional inhibition of MHC-I genes, thereby inducing tumor cells to evade CD8^+^ T cell-mediated immunosurveillance.

## 2 Results

### 2.1 Nuclear GLDC is polyubiquitinated at K636 upon EGFR activation

**Fig. 1:**
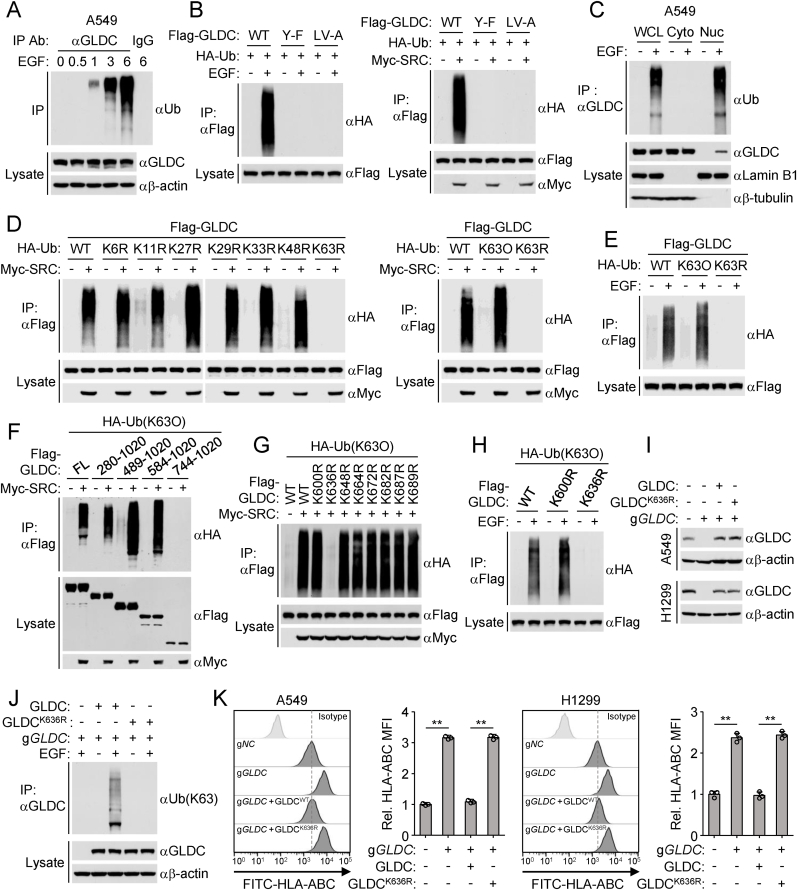
**Nuclear GLDC is polyubiquitinated at K636 after EGFR activation.** (A) EGF treatment induces polyubiquitination of GLDC. A549 cells were serum-starved (12 h) and then treated with or without EGF (100 ng/mL) for the indicated times before co-immunoprecipitation and immunoblotting analysis with the indicated antibodies. (B) Effects of GLDC mutations on the polyubiquitination of GLDC. HEK293/EGFR cells were transfected with the indicated plasmids for 24 h and then treated with or without EGF (100 ng/mL) for 6 h. HEK293 cells were transfected with the indicated plasmids for 24 h. The cells were collected for co-immunoprecipitation and immunoblotting analysis with the indicated antibodies. (C) EGF induces polyubiquitination of GLDC in nucleus. A549 cells were serum-starved (12 h) and then treated with or without EGF (100 ng/mL) for 6 h before subcellular fractionation experiments. Subcellular fractions were co-immunoprecipitated and analyzed by immunoblotting with the indicated antibodies. (D) SRC induces K63-linked polyubiquitination of GLDC. HEK293 cells were transfected with the indicated plasmids for 24 h before co-immunoprecipitation and immunoblotting analysis with the indicated antibodies. (E) EGF induces K63-linked polyubiquitination of GLDC. HEK293/EGFR cells were transfected with the indicated plasmids for 24 h and then treated with or without EGF (100 ng/mL) for 6 h before co-immunoprecipitation and immunoblotting analysis with the indicated antibodies. (F) Effects of GLDC truncation mutants on SRC-induced K63-linked polyubiquitination of GLDC. HEK293 cells were transfected with the indicated plasmids for 24 h before co-immunoprecipitation and immunoblotting analysis with the indicated antibodies. (G) Effects of GLDC mutants on SRC-induced K63-linked polyubiquitination of GLDC. HEK293 cells were transfected with the indicated plasmids for 24 h before co-immunoprecipitation and immunoblotting analysis with the indicated antibodies. (H) Effects of GLDC mutants on EGF-induced K63-linked polyubiquitination of GLDC. HEK293/EGFR cells were transfected with the indicated plasmids for 24 h and then treated with or without EGF (100 ng/mL) for 6 h before co-immunoprecipitation and immunoblotting analysis with the indicated antibodies. (I) Reconstitution of GLDC-deficient cells with wild-type GLDC or GLDC^K636R^. Lysates of the indicated cells were analyzed by immunoblots with the indicated antibodies. (J) EGF stimulation induces K63-linked polyubiquitination of wild-type GLDC but not GLDC^K636R^. Reconstitution of GLDC-deficient A549 cells with wild-type GLDC or GLDC^K636R^. The indicated cells were serum-starved (12 h) and then treated with or without EGF (100 ng/mL) for 6 h before co-immunoprecipitation and immunoblotting analysis with the indicated antibodies. (K) Effects of GLDC mutations on MHC-I surface expression. Control (g*NC*) or GLDC-deficient (g*GLDC*) A549 or H1299 cells were reconstituted with human wild-type GLDC or GLDC^K636R^. The cells were cultured in the presence of EGF (50 ng/mL) for 24 h before flow cytometry analysis with the indicated antibodies. Graph shows mean ± SEM, *n* = 3 independent samples. Data were analyzed using two-way ANOVA with GraphPad Prism 8. ^∗∗^*P* < 0.01.

Cytosolic GLDC is phosphorylated by SRC following EGFR activation and subsequently translocated into the nucleus. Nuclear GLDC acts as a co-repressor of STAT1 to suppress STAT1-triggered MHC-I expression. To explore the mechanisms how nuclear GLDC regulates STAT1-triggered MHC-I expression, we analyzed the posttranslational modifications of GLDC in response to EGF treatment. Time-course experiments showed that EGF treatment induced GLDC polyubiquitination, and the modification levels became detectable within 1 h and remained at a higher level during prolonged EGF stimulation (Fig. 1(A)). Our previous studies have demonstrated that EGF stimulation triggers SRC-mediated phosphorylation of GLDC at Y993 and Y1008, and GLDC^Y993F/Y1008F^ fails to be phosphorylated and translocated into the nucleus. L998/V999 is also important for GLDC nuclear translocation following EGF treatment, and GLDC^L998A/V999A^ failed to be translocated into the nucleus. Here, we found that EGF treatment or overexpression of SRC induced polyubiquitination of wild-type GLDC but not GLDC^Y993F/Y1008F^ (GLDC^Y−F^) or GLDC^L998A/V999A^ (GLDC^LV−A^) (Fig. 1(B)), suggesting that phosphorylation and nuclear translocation of GLDC is required for its polyubiquitination. Further cellular fractionation experiments identified that EGF stimulation induced polyubiquitination of GLDC in the nucleus (Fig. 1(C)).

We next investigated the molecular mechanisms of EGF-mediated polyubiquitination of GLDC. Utilizing ubiquitin mutants in which one or six lysine residues are replaced with arginine (R), we found that overexpression of SRC increased K63- but not other lysine residue-linked polyubiquitination of GLDC (Fig. 1(D)). Consistently, EGF treatment induced K63-linked polyubiquitination of GLDC (Fig. 1(E)). To further identify the residues in GLDC that are conjugated with K63-linked polyubiquitin chains following EGF stimulation, we analyzed which parts of GLDC are polyubiquitinated after overexpression of SRC. Domain mapping experiments revealed that overexpression of SRC induced K63-linked polyubiquitination of wild-type GLDC, GLDC(280-1020), GLDC(489-1020) or GLDC(584-1020) but not GLDC(744-1020) (Fig. 1(F)), suggesting that the aa584-743 of GLDC is important for its polyubiquitination induced by overexpression of SRC. We then individually mutated each of the 8 lysine residues within aa584-743 of GLDC to arginine and examined whether the mutants could be modified by K63-linked polyubiquitination. The results indicated that mutation of K636 but not the other 7 lysine residues in GLDC to arginine dramatically impaired its K63-linked polyubiquitination (Fig. 1(G)). Sequence analysis showed that K636 of GLDC was conserved in various vertebrate species (Fig. S1). Consistently, EGF stimulation induced K63-linked polyubiquitination of wild-type GLDC and GLDC^K600R^ but not GLDC^K636R^ (Fig. 1(H)). We next reconstituted GLDC-knockout A549 cells with wild-type GLDC or GLDC^K636R^ and found that EGF stimulation increased K63-linked polyubiquitination of wild-type GLDC but not GLDC^K636R^ (Fig. 1(I) and (J)). Taken together, these results suggest that EGF induces GLDC^K636^ for K63-linked polyubiquitination.

To determine whether K63-linked polyubiquitination of GLDC regulates MHC-I expression, we examined the MHC-I levels in wild-type GLDC or GLDC^K636R^-reconstituted GLDC-deficient A549 or H1299 cells. Flow cytometry analysis showed that reconstitution of wild-type GLDC but not GLDC^K636R^ abrogated the up-regulation of MHC-I in GLDC-deficient cells (Fig. 1(K)). These results suggest that K63-linked polyubiquitination of GLDC at K636 inhibits MHC-I surface expression.

### 2.2 FBXL3 mediates K63-linked polyubiquitination of GLDC

**Fig. 2:**
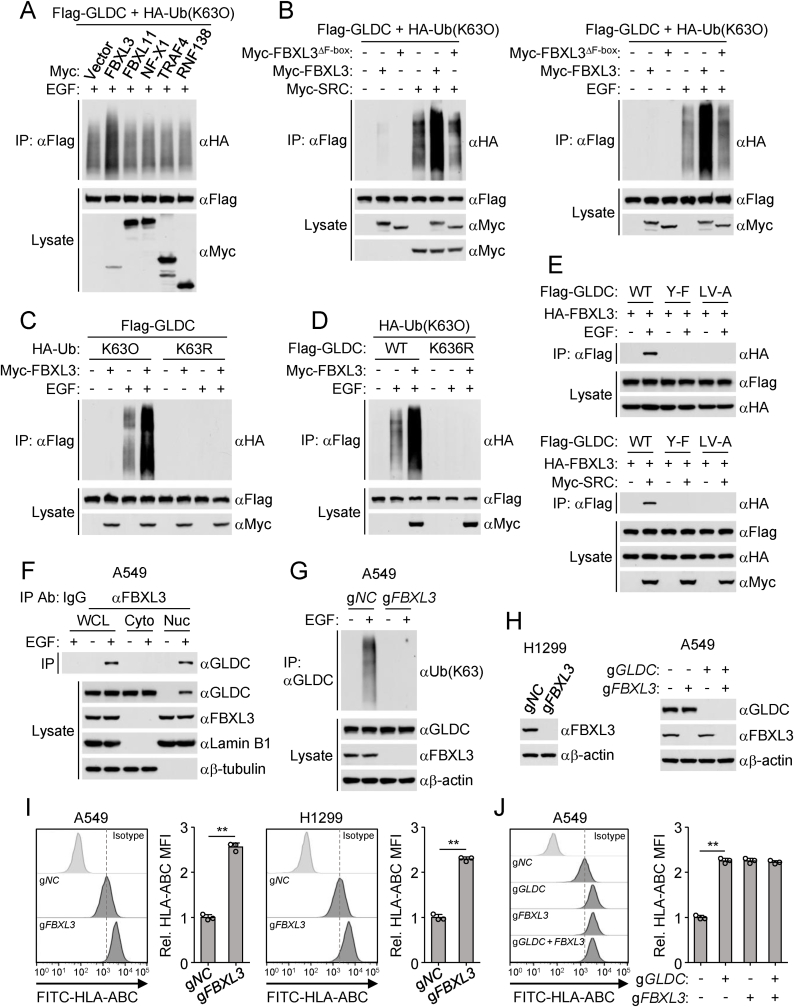
**FBXL3 mediates K63 linked-polyubiquitination of GLDC.** (A) FBXL3 promotes polyubiquitination of GLDC. HEK293/EGFR cells were transfected with the indicated plasmids for 24 h and then treated with EGF (100 ng/mL) for 6 h before co-immunoprecipitation and immunoblotting analysis with the indicated antibodies. (B) FBXL3 but not its inactive mutant FBXL3^ΔF-box^ promotes K63-linked polyubiquitination of GLDC. HEK293 cells were transfected with the indicated plasmids for 24 h (left). HEK293/EGFR cells were transfected with the indicated plasmids for 24 h and then treated with or without EGF (100 ng/mL) for 6 h (right). The cells were collected for co-immunoprecipitation and immunoblotting analysis with the indicated antibodies. (C) FBXL3 promotes EGF-induced K63-linked polyubiquitination of GLDC. HEK293/EGFR cells were transfected with the indicated plasmids for 24 h and then treated with or without EGF (100 ng/mL) for 6 h before co-immunoprecipitation and immunoblotting analysis with the indicated antibodies. (D) FBXL3 increases K63-linked polyubiquitination of wild-type GLDC but not GLDC^K636R^. HEK293/EGFR cells were transfected with the indicated plasmids for 24 h and then treated with or without EGF (100 ng/mL) for 6 h before co-immunoprecipitation and immunoblotting analysis with the indicated antibodies. (E) Effects of GLDC mutations on the interaction of FBXL3 with GLDC. HEK293/EGFR cells were transfected with the indicated plasmids for 24 h and then treated with or without EGF (100 ng/mL) for 6 h (up). HEK293 cells were transfected with the indicated plasmids for 24 h (down). The cells were collected for co-immunoprecipitation and immunoblotting analysis with the indicated antibodies. (F) EGF induces the interaction of GLDC with FBXL3 in nucleus. A549 cells were serum-starved (12 h) and then treated with or without EGF (100 ng/mL) for 6 h before subcellular fractionation experiments. Subcellular fractions were co-immunoprecipitated and analyzed by immunoblotting with the indicated antibodies. (G) Knockout of FBXL3 inhibits EGF-induced polyubiquitination of GLDC. Control (g*NC*) or FBXL3-deficient (g*FBXL3)* A549 cells were serum-starved (12 h) and then treated with or without EGF (100 ng/mL) for 6 h before co-immunoprecipitation and immunoblotting analysis with the indicated antibodies. (H–J) FBXL3-deficiency increases MHC-I surface expression. The indicated cells were cultured in the presence of EGF (50 ng/mL) for 24 h before flow cytometry analysis with the indicated antibodies. Graph shows mean ± SEM, *n* = 3 independent samples. Data were analyzed using two-way ANOVA with GraphPad Prism 8. ^∗∗^*P* < 0.01.

To identify the E3 ubiquitin ligases responsible for K63-linked polyubiquitination of GLDC, we performed a screen of 196 ubiquitin-related proteins for their abilities to regulate GLDC polyubiquitination using co-transfection assays in HEK293/EGFR cells. These efforts led to the identification of FBXL3, which promoted K63-linked polyubiquitination of GLDC (Fig. 2(A)). Overexpression of FBXL3 but not its inactive mutant FBXL3^ΔF-box^ promoted K63-linked polyubiquitination of GLDC induced by SRC overexpression or EGF treatment (Fig. 2(B)). And FBXL3 increased K63- but not other lysine residue-linked polyubiquitination of GLDC (Fig. 2(C)). FBXL3 enhanced K63-linked polyubiquitination of wild-type GLDC but not GLDC^K636R^ following EGF stimulation (Fig. 2(D)). Co-immunoprecipitation experiments indicated that FBXL3 was associated with wild-type GLDC but not GLDC^Y−F^ or GLDC^LV−A^ after EGF treatment or overexpression of SRC (Fig. 2(E)). Endogenous co-immunoprecipitation further indicated that GLDC was associated with FBXL3 in the nucleus (Fig. 2(F)). Confocal microscopy further confirmed that GLDC was co-located with FBXL3 in the nucleus after EGF stimulation (Fig. S2). FBXL3-deficiency abolished the K63-linked polyubiquitination of GLDC induced by EGF stimulation (Fig. 2(G)). Collectively, these results suggest that FBXL3 mediates K63-linked polyubiquitination of GLDC at K636 following EGF stimulation in the nucleus. In addition, FBXL3-deficiency increased MHC-I surface expression in A549 or H1299 cells (Fig. 2(H) and (I)). Notably, knockout of GLDC abolished up-regulation of MHC-I induced by FBXL3-deficiency (Fig. 2 (H) and (J)). These results suggest that FBXL3 regulates MHC-I expression through GLDC.

### 2.3 USP22 antagonizes FBXL3-mediated GLDC polyubiquitination

**Fig. 3:**
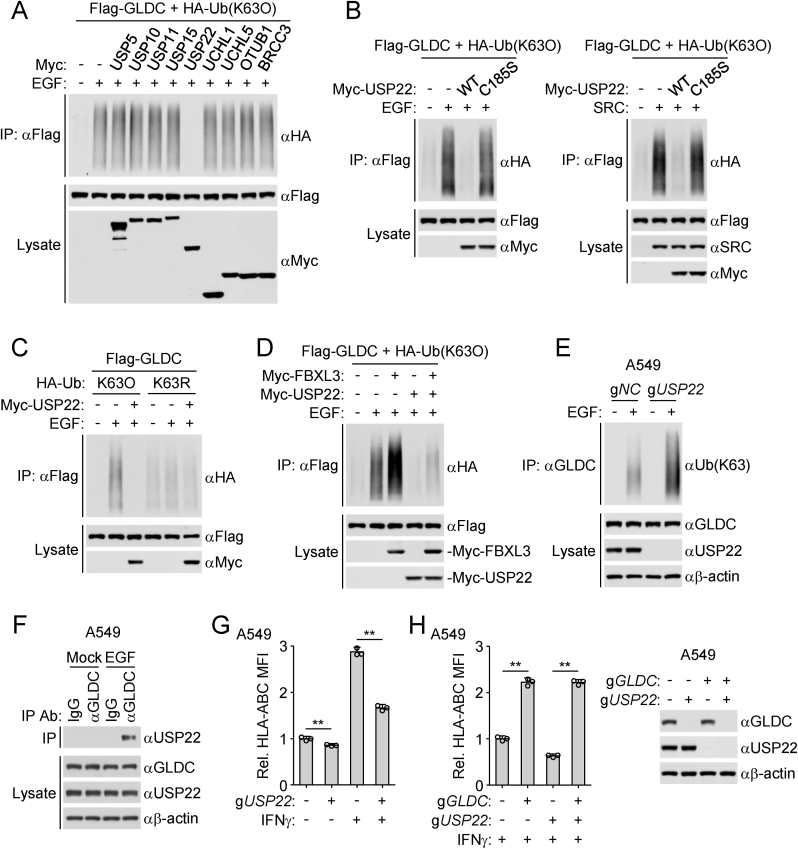
**USP22 antagonizes FBXL3-mediated GLDC polyubiquitination.** (A) USP22 removes K63-linked polyubiquitin moieties from GLDC. HEK293/EGFR cells were transfected with the indicated plasmids for 24 h and then treated with or without EGF (100 ng/mL) for 6 h before co-immunoprecipitation and immunoblotting analysis with the indicated antibodies. (B) USP22 but not its inactive mutant USP22^C185S^ removes K63-linked polyubiquitin moieties from GLDC. HEK293/EGFR cells were transfected with the indicated plasmids for 24 h and then treated with or without EGF (100 ng/mL) for 6 h (left). HEK293 cells were transfected with the indicated plasmids for 24 h (right). The cells were collected for co-immunoprecipitation and immunoblotting analysis with the indicated antibodies. (C) USP22 removes K63- but not other lysine residue-linked polyubiquitination of GLDC. HEK293/EGFR cells were transfected with the indicated plasmids for 24 h and then treated with or without EGF (100 ng/mL) for 6 h before co-immunoprecipitation and immunoblotting analysis with the indicated antibodies. (D) USP22 removes K63-linked polyubiquitin moieties of GLDC catalyzed by FBXL3. HEK293/EGFR cells were transfected with the indicated plasmids for 24 h and then treated with or without EGF (100 ng/mL) for 6 h before co-immunoprecipitation and immunoblotting analysis with the indicated antibodies. (E) Knockout of USP22 enhances EGF-induced polyubiquitination of GLDC. Control (g*NC*) or USP22-deficient (g*USP22)* A549 cells were serum-starved (12 h) and then treated with or without EGF (100 ng/mL) for 6 h before co-immunoprecipitation and immunoblotting analysis with the indicated antibodies. (F) EGF induces the interaction of GLDC with USP22. A549 cells were serum-starved (12 h) and then treated with or without EGF (100 ng/mL) for 6 h before co-immunoprecipitated and analyzed by immunoblotting with the indicated antibodies. (G and H) FBXL3-deficiency inhibits MHC-I surface expression. The indicated cells were cultured in the presence of EGF (50 ng/mL) for 24 h and then stimulated with IFNγ (50 ng/mL) for 24 h before flow cytometry analysis with the indicated antibodies. Graph shows mean ± SEM, *n* = 3 independent samples. Data were analyzed using two-way ANOVA with GraphPad Prism 8. ^∗∗^*P* < 0.01.

We next attempted to identify deubiquitinating enzymes that specifically reverse K63-linked polyubiquitin of GLDC. GLDC-bound proteins were immunoprecipitated with anti-GLDC antibody and analyzed by mass spectrometry. From the resulting interactome data, we identified 9 candidate deubiquitinating enzymes (Table S1). Co-transfection experiments indicated that only USP22 but not the other 8 enzymes removed K63-linked polyubiquitin moieties from GLDC induced by EGF stimulation in HEK293/EGFR cells (Fig. 3(A)). USP22 but not its enzymatic inactive mutant USP22^C185S^ removed K63-linked polyubiquitin moieties from GLDC induced by EGF stimulation or overexpression of SRC (Fig. 3(B)). USP22 removed K63- but not other lysine residue-linked polyubiquitination of GLDC (Fig. 3(C)). In addition, USP22 removed K63-linked polyubiquitination of GLDC catalyzed by FBXL3 (Fig. 3(D)). Endogenous ubiquitination assays indicated that EGF stimulation induced K63-linked polyubiquitination of GLDC, which was enhanced in USP22-deficiency A549 cells (Fig. 3(E)). Co-immunoprecipitation experiments indicated that EGF stimulation induced the interaction of GLDC with USP22 (Fig. 3(F)). In addition, USP22-deficiency down-regulated the levels of MHC-I in A549 (Fig. 3(G)). Notably, knockout of USP22 failed to down-regulation of MHC-I in GLDC-deficient A549 cells (Fig. 3(H)). Taken together, these results suggest that USP22 regulates MHC-I expression through GLDC.

### 2.4 EGFR activation triggers SRC-mediated phosphorylation of FBXL3^Y306^

**Fig. 4:**
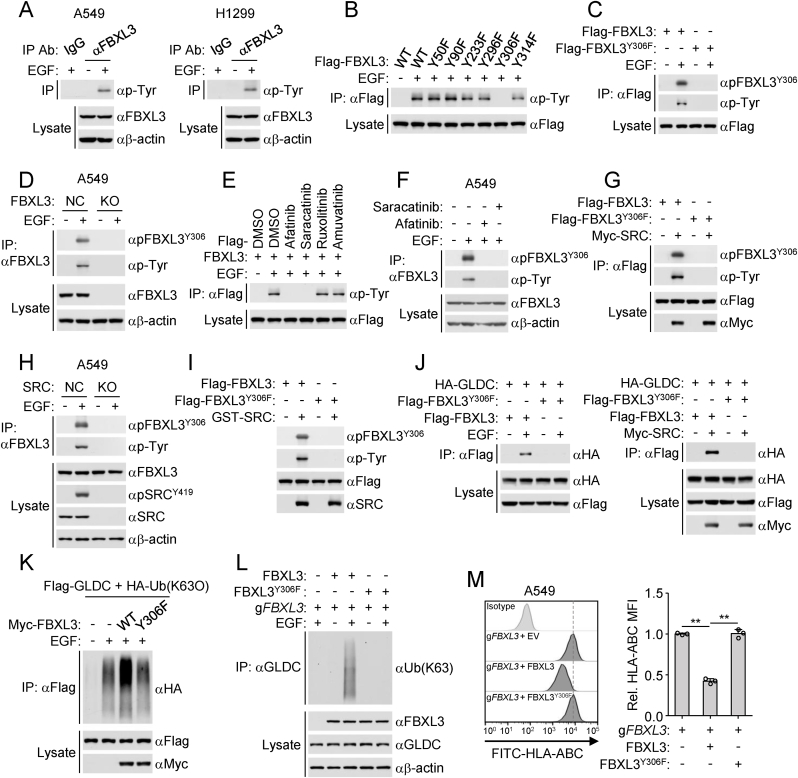
**EGFR activation triggers SRC-mediated phosphorylation of FBXL3**^**Y306**^**.** (A) EGF stimulation induces FBXL3 tyrosine phosphorylation. A549 or H1299 cells were serum-starved (12 h) and then treated with or without EGF (100 ng/mL) for 6 h before co-immunoprecipitation and immunoblotting analysis with the indicated antibodies. (B and C) Effects of FBXL3 mutations on EGF-induced phosphorylation of FBXL3. HEK293/EGFR cells were transfected with the indicated plasmids for 24 h and then treated with or without EGF (100 ng/mL) for 6 h before co-immunoprecipitation and immunoblotting analysis with the indicated antibodies. (D) FBXL3 is phosphorylated following EGF stimulation. Control (g*NC*) or FBXL3-deficient (g*FBXL3)* A549 cells were serum-starved (12 h) and then treated with or without EGF (100 ng/mL) for 6 h before co-immunoprecipitation and immunoblotting analysis with the indicated antibodies. (E and F) Afatinib or Saracatinib treatment inhibits EGF-induced phosphorylation of FBXL3^Y306^. HEK293/EGFR cells were transfected with the indicated plasmids for 24 h and then treated with Afatinib (2 μM), Saracatinib (1 μM), Ruxolitinib(5 μM) or Amuvatinib(5 μM) for 2 h before EGF (100 ng/mL) treatment for 6 h (E). A549 cells were serum-starved (12 h) and then treated with Afatinib (2 μM) or Saracatinib (1 μM) for 2 h before EGF (100 ng/mL) treatment for 6 h (F). The cells were collected for co-immunoprecipitation and immunoblotting analysis with the indicated antibodies. (G) Overexpression of SRC induces phosphorylation of FBXL3. HEK293 cells were transfected with the indicated plasmids for 24 h before co-immunoprecipitation and immunoblotting analysis with the indicated antibodies. (H) Knockout of SRC inhibits EGF-induced phosphorylation of FBXL3. Control (g*NC*) or SRC-deficient (g*SRC)* A549 cells were serum-starved (12 h) and then treated with or without EGF (100 ng/mL) for 6 h before co-immunoprecipitation and immunoblotting analysis with the indicated antibodies. (I) SRC mediates FBXL3 phosphorylation *in vitro*. The indicated recombinant proteins were incubated for *in vitro* kinase assays before immunoblotting analysis with the indicated antibodies. (J) FBXL3^Y306F^ fails to interact with GLDC following EGF treatment or overexpression of SRC. HEK293/EGFR cells were transfected with the indicated plasmids for 24 h and then treated with or without EGF (100 ng/mL) for 6 h. HEK293 cells were transfected with the indicated plasmids for 24 h. The cells were collected for co-immunoprecipitation and immunoblotting analysis with the indicated antibodies. (K) FBXL3^Y306F^ fails to increase K63-linked polyubiquitination of GLDC. HEK293/EGFR cells were transfected with the indicated plasmids for 24 h and then treated with or without EGF (100 ng/mL) for 6 h before co-immunoprecipitation and immunoblotting analysis with the indicated antibodies. (L) Effects of FBXL3 mutation on EGF-induced K63-linked polyubiquitination of GLDC. Reconstitution of FBXL3-deficient A549 cells with wild-type FBXL3 or FBXL3^Y306F^. The indicated cells were serum-starved (12 h) and then treated with or without EGF (100 ng/mL) for 6 h before co-immunoprecipitation and immunoblotting analysis with the indicated antibodies. (M) Effects of FBXL3 mutation on MHC-I surface expression. Reconstitution of FBXL3-deficient A549 cells with wild-type FBXL3 or FBXL3^Y306F^. The cells were cultured in the presence of EGF (50 ng/mL) for 24 h before flow cytometry analysis with the indicated antibodies. Graph shows mean ± SEM, *n* = 3 independent samples. Data were analyzed using two-way ANOVA with GraphPad Prism 8. ^∗∗^*P* < 0.01.

In our experiments, we demonstrated that FBXL3-mediated K63-linked polyubiquitination of GLDC is required for GLDC to inhibit MHC-I expression. Next, we explored how FBXL3 targets GLDC polyubiquitination. Immunoblotting analysis indicated that EGF stimulation induced tyrosine phosphorylation of FBXL3 (Fig. 4(A)). We individually mutated each of the 6 tyrosine residues of FBXL3, Y50, Y90, Y233, Y296, Y306 and Y314, to phenylalanine and examined whether these mutants could be modified by phosphorylation. The results indicated that mutations of Y306 but not the other 5 tyrosine residues to phenylalanine impaired EGF-induced tyrosine phosphorylation of FBXL3 (Fig. 4(B)). Sequence analysis indicated that FBXL3^Y306^ was conserved in various vertebrate species (Fig. S3). To determine whether EGF mediates phosphorylation of FBXL3^Y306^, we generated a rabbit polyclonal antibody specific for Y306-phosphorylated FBXL3 (pFBXL3^Y306^). Immunoblotting analysis confirmed that FBXL3^Y306^ was phosphorylated following EGF stimulation, and EGF stimulation failed to induce FBXL3^Y306F^ phosphorylation (Fig. 4(C) and (D)). These results suggest that EGF mediates phosphorylation of FBXL3 at Y306.

To identify the tyrosine kinases responsible for FBXL3 phosphorylation upon EGFR activation, we employed a targeted inhibitor approach in HEK293/EGFR cells. Cells were treated with four kinase inhibitors: Afatinib (EGFR inhibitor), Saracatinib (SRC family kinase inhibitor), Ruxolitinib (JAK family kinase inhibitor) and Amuvatinib (c-Kit inhibitor). Immunoblotting analysis showed that Afatinib or Saracatinib treatment dramatically impaired phosphorylation of FBXL3 induced by EGF stimulation (Fig. 4(E)). Further confirmative experiments identified that both Afatinib and Saracatinib treatment abrogated EGF-induced phosphorylation of FBXL3^Y306^ in A549 cells (Fig. 4(F)). In addition, overexpression of SRC induced tyrosine phosphorylation of wild-type FBXL3 but not FBXL3^Y306F^ (Fig. 4(G)). SRC-deficiency impaired EGF-induced FBXL3^Y306^ phosphorylation (Fig. 4(H)). *In vitro* phosphorylation assays further showed that SRC catalyzed tyrosine phosphorylation of wild-type FBXL3, but not FBXL3^Y306F^ (Fig. 4(I)). These results suggest that SRC catalyzes phosphorylation of FBXL3 at Y306.

We next investigated the function of FBXL3^Y306^ phosphorylation. Co-immunoprecipitation experiments indicated that EGF stimulation or overexpression of SRC induced the interaction of GLDC with wild-type FBXL3, but not FBXL3^Y306F^ (Fig. 4(J)). Overexpression of FBXL3 but not FBXL3^Y306F^ promoted K63-linked polyubiquitination of GLDC induced by EGF treatment (Fig. 4(K)). Next, we reconstituted FBXL3-knockout A549 cells with wild-type FBXL3 or FBXL3^Y306F^ and found that EGF stimulation enhanced K63-linked polyubiquitination of GLDC in wild-type FBXL3-reconstituted A549 cells but not in FBXL3^Y306F^-reconstituted A549 cells (Fig. 4(L)). Taken together, these results suggest that SRC-phosphorylated-FBXL3 mediates GLDC polyubiquitination. In addition, reconstitution of wild-type FBXL3 but not FBXL3^Y306F^ abrogated the up-regulation of MHC-I in FBXL3-deficient A549 cells (Fig. 4(M)), suggesting that phosphorylation of FBXL3^Y306^ inhibits MHC-I expression.

### 2.5 GLDC polyubiquitination promotes the recruitment of SMARCE1 and DMAP1

**Fig. 5:**
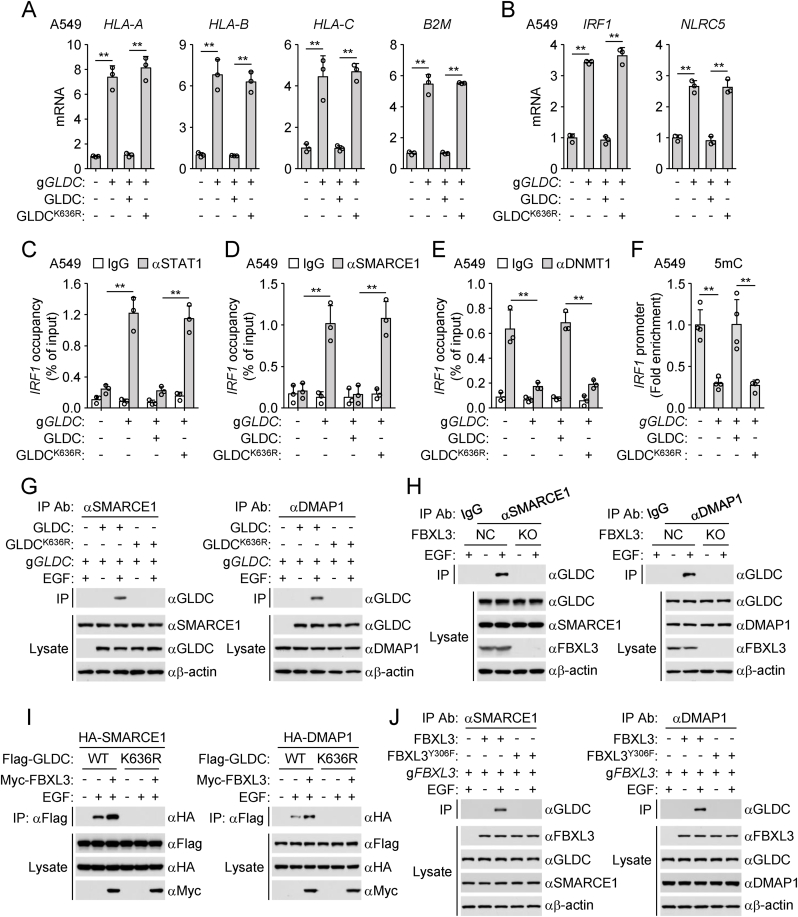
**GLDC polyubiquitination promotes the recruitment of SMARCE1 and DMAP1.** (A and B) Effects of GLDC mutations on the transcription of *HLA-A*, *HLA-B*, *HLA-C*, *B2M*, *IRF1* and *NLRC5* genes. Control (g*NC*) or GLDC-deficient (g*GLDC*) A549 cells were reconstituted with human wild-type GLDC or GLDC^K636R^. The cells were cultured in the presence of EGF (50 ng/mL) for 24 h before qPCR analysis of mRNA levels of the indicated genes. Graph shows mean ± SEM, *n* = 3 independent samples. Data were analyzed using two-way ANOVA with GraphPad Prism 8. ^∗∗^*P* < 0.01. (C–E) Effects of GLDC mutations on the binding of STAT1, SMARCE1 or DNMT1 to *IRF1* promoter region. Control (g*NC*) or GLDC-deficient (g*GLDC*) A549 cells were reconstituted with human wild-type GLDC or GLDC^K636R^. The cells were cultured in the presence of EGF (50 ng/mL) for 24 h before ChIP analysis. The de-crosslinked DNA was subjected to qPCR analysis using specific primers. Graph shows mean ± SEM, *n* = 3 independent samples. Data were analyzed using two-way ANOVA with GraphPad Prism 8. ^∗∗^*P* < 0.01. (F) Effects of GLDC mutations on the methylation status of *IRF1* promoter region. Control (g*NC*) or GLDC-deficient (g*GLDC*) A549 cells were reconstituted with human wild-type GLDC or GLDC^K636R^. The cells were cultured in the presence of EGF (50 ng/mL) for 24 h before MeDIP analysis. The de-crosslinked DNA was subjected to qPCR analysis using specific primers. Graph shows mean ± SEM, *n* = 4 independent samples. Data were analyzed using two-way ANOVA with GraphPad Prism 8. ^∗∗^*P* < 0.01. (G) Effects of GLDC mutations on the interaction of SMARCE1 or DMAP1 with GLDC. Control (g*NC*) or GLDC-deficient (g*GLDC*) A549 cells were reconstituted with human wild-type GLDC or GLDC^K636R^. The cells were serum-starved (12 h) and then treated with or without EGF (100 ng/mL) for 6 h before co-immunoprecipitation and immunoblotting analysis with the indicated antibodies. (H) Effects of FBXL3-deficiency on the interaction of SMARCE1 or DMAP1 with GLDC. Control (g*NC*) or FBXL3-deficient (g*FBXL3*) A549 cells were serum-starved (12 h) and then treated with or without EGF (100 ng/mL) for 6 h before co-immunoprecipitation and immunoblotting analysis with the indicated antibodies. (I) Overexpression of FBXL3 promotes the interaction of SMARCE1 or DMAP1 with wild-type GLDC but not GLDC^K636R^. HEK293/EGFR cells were transfected with the indicated plasmids for 24 h and then treated with or without EGF (100 ng/mL) for 6 h before co-immunoprecipitation and immunoblotting analysis with the indicated antibodies. (J) Effects of FBXL3 mutations on the interaction of SMARCE1 or DMAP1 with GLDC. Reconstitution of FBXL3-deficient A549 cells with wild-type FBXL3 or FBXL3^Y306F^. The cells were serum-starved (12 h) and then treated with or without EGF (100 ng/mL) for 6 h before co-immunoprecipitation and immunoblotting analysis with the indicated antibodies.

Our previous studies have demonstrated that upon EGFR activation, the nuclear GLDC hijacks the STAT1 co-activator SMARCE1 to inhibit the binding of STAT1 to promoter regions of *IRF1* and *NLRC5* genes, and the GLDC/SMARCE1/STAT1 complex also recruits DMAP1/DNMT1 to induce promoter DNA hypermethylation of *IRF1* and *NLRC5* genes, resulting in transcriptional inhibition of the downstream MHC-I genes. Quantitative real-time PCR (qPCR) experiments showed that GLDC-deficiency up-regulated the mRNA levels of MHC-I genes, including *HLA-A*, *HLA-B*, *HLA-C* and *B2M*, which was reversed by reconstitution with wild-type GLDC but not GLDC^K636R^ (Fig. 5(A)). Reconstitution of wild-type GLDC but not GLDC^K636R^ abrogated up-regulation of the mRNA levels of *IRF1* and *NLRC5* genes in GLDC-deficient cells (Fig. 5(B)). GLDC-deficiency enhanced the binding of STAT1 and SMARCE1 to *IRF1* promoter region, which was reversed by reconstitution with wild-type GLDC but not GLDC^K636R^ (Fig. 5(C) and (D)). The reduced DNMT1 binding to *IRF1* promoter region in GLDC-deficient cells was restored by reconstitution with wild-type GLDC but not GLDC^K636R^ (Fig. 5(E)). MeDIP also showed that reconstitution of wild-type GLDC but not GLDC^K636R^ abrogated down-regulation of the levels of *IRF1* promoter DNA methylation in GLDC-deficient cells (Fig. 5(F)). These results suggest that polyubiquitination of GLDC^K636^ inhibits STAT1-triggered transcriptional activation.

We next investigated the function of GLDC^K636^ polyubiquitination. Co-immunoprecipitation experiments showed that SMARCE1 and DMAP1 was associated with wild-type GLDC but not GLDC^K636R^ after EGF stimulation (Fig. 5(G)), suggesting that K63-linked polyubiquitination of GLDC^K636^ is required for its recruitment of SMARCE1 and DMAP1. Consistently, FBXL3-deficiency abolished the interaction of GLDC with SMARCE1 and DMAP1 following EGF treatment (Fig. 5(H)). Overexpression of FBXL3 enhanced the interaction of SMARCE1 and DMAP1 with wild-type GLDC but not GLDC^K636R^ (Fig. 5(I)). Additionally, reconstitution of wild-type FBXL3 but not FBXL3^Y306F^ in FBXL3-deficient cells restored the interaction of SMARCE1 and DMAP1 with GLDC (Fig. 5(J)). Together, the binding of GLDC with SMARCE1 and DMAP1 is strictly dependent on FBXL3-mediated K63-linked polyubiquitination of GLDC^K636^.

### 2.6 Polyubiquitination of GLDC^K636^ inhibits CD8^+^ T cell-mediated immunosurveillance

**Fig. 6:**
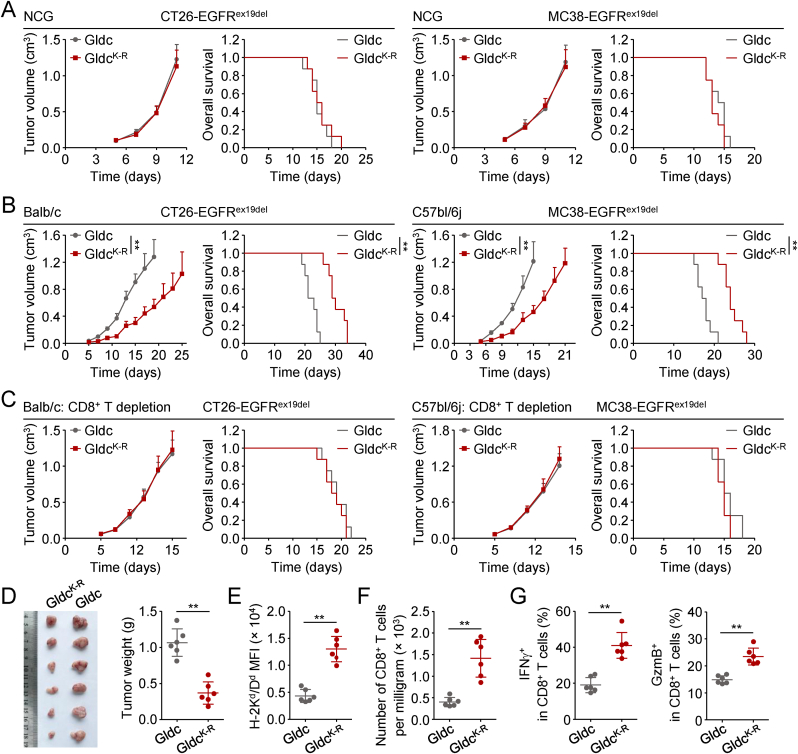
**Polyubiquitination of GLDC**^**K636**^**inhibits CD8**^**+**^**T cell-mediated immunosurveillance.** (A–C) Effects of GLDC mutation on tumor growth. Gldc-deficient (g*Gldc*) CT26-EGFR^ex19del^ or MC38-EGFR^ex19del^ cells (5 × 10^5^) reconstituted with mouse wild-type Gldc or Gldc^K641R^ (Gldc^K−R^) were subcutaneously injected into NCG mice. On day 5 after tumor cell inoculation, tumor sizes were measured every two days by caliper. Mice were sacrificed when the tumor size was bigger than 15 mm of the mean tumor diameter, tumor volume exceeded 2000 mm^3^, or tumor had ulcers with diameter reached 10 mm. Graph shows mean ± SEM, *n* = 8 (A), *n* = 8 (B), *n* = 9 (C). Data were analyzed using two-way ANOVA with GraphPad Prism 8. Kaplan–Meier survival curves and corresponding log-rank (Mantel-Cox) tests were used to evaluate the statistical differences between groups in survival studies. ^∗∗^*P* < 0.01. (D) GLDC^K636R^ inhibits tumor growth. Gldc-deficient (g*Gldc*) CT26-EGFR^ex19del^ cells (5 × 10^5^) reconstituted with mouse wild-type Gldc or Gldc^K641R^ (Gldc^K−R^) were subcutaneously injected into Balb/c mice. Tumor-bearing mice were euthanized on day 18, and then tumor tissues were separated from the mice. Tumor weights were measured by Analytical Balance. Graph shows mean ± SEM, *n* = 6. Data were analyzed using a student's unpaired *t*-test with GraphPad Prism 8. ^∗∗^*P* < 0.01. (E) Effects of GLDC mutations on MHC-I surface expression of tumor cells *in vivo*. Gldc-deficient (g*Gldc*) CT26-EGFR^ex19del^ cells (5 × 10^5^) reconstituted with mouse wild-type Gldc or Gldc^K641R^ (Gldc^K−R^) were subcutaneously injected into Balb/c mice. Tumor-bearing mice were euthanized on day 16, and then tumor tissues were separated from the mice. Tumor cells were isolated from the indicated tumor tissues, stained with the indicated antibodies and analyzed by flow cytometry. Graph shows mean ± SEM, *n* = 6 independent samples. Data were analyzed using a student's unpaired *t*-test with GraphPad Prism 8. ^∗∗^*P* < 0.01. (F) Effects of GLDC mutations on the numbers of CD8^+^ T cells in TME. TILs were isolated from the CT26-EGFR^ex19del^ tumor tissues in Fig. 6(D). TILs were stained with the indicated antibodies and analyzed by flow cytometry. CD8^+^ T cells were sorted from CD45^+^ populations. Graph shows mean ± SEM, *n* = 6 independent samples. Data were analyzed using a student's unpaired *t*-test with GraphPad Prism 8. ^∗∗^*P* < 0.01. (G) GLDC^K636R^ increases tumor infiltrating CD8^+^ cytotoxic T cells. TILs were isolated from the CT26-EGFR^ex19del^ tumor tissues in Fig. 6(D). TILs were stained with the indicated antibodies and analyzed by flow cytometry. Graph shows mean ± SEM, *n* = 6 independent samples. Data were analyzed using a student's unpaired *t*-test with GraphPad Prism 8. ^∗∗^*P* < 0.01.

We next investigated the biological functions of GLDC^K636^ polyubiquitination. The EGFR^ex19del^ mutation, characterized by an in-frame deletion of five amino acids (Glu746-Ala750) in exon 19 of EGFR gene, represents a canonical activating mutation that confers constitutive kinase activity and oncogenic potential. We transduced the mouse tumor cells (CT26 and MC38) with EGFR^ex19del^ to mimic the constitutively EGFR activation *in vitro*. Flow cytometry analysis showed that reconstitution of wild-type Gldc but not mouse Gldc^K641R^ (Gldc^K−R^, in human is GLDC^K636^) abrogated the up-regulation of MHC-I in GLDC-deficient CT26-EGFR^ex19del^ or MC38-EGFR^ex19del^ cells (Fig. S4A). Cell proliferation assays showed that reconstitution of GLDC-deficient CT26-EGFR^ex19del^ or MC38-EGFR^ex19del^ cells with mouse Gldc^K−R^ promoted cell proliferation to a similar degree comparing to reconstitution with wild-type Gldc (Fig. S4B). Consistently, tumor growth and animal survival in NCG mice subcutaneously injected with Gldc^K−R^-reconstituted CT26-EGFR^ex19del^ or MC38-EGFR^ex19del^ cells did not show significant difference in comparison to those injected with wild-type Gldc-reconstituted cells (Fig. 6(A)). These results suggest that polyubiquitination of GLDC^K636^ did not affect tumor cell proliferation. Next, we subcutaneously injected the wild-type Gldc or Gldc^K−R^-reconstituted CT26-EGFR^ex19del^ or MC38-EGFR^ex19del^ cells in immune-competent Balb/c or C57bl/6j mice respectively, and found that reconstitution of Gldc^K−R^ in these cells suppressed tumor growth and improved the overall survival compared to reconstitution of wild-type Gldc in these cells (Fig. 6(B)). Depletion of CD8^+^ T cells completely abrogated the tumor-suppressive function induced by reconstitution of Gldc^K−R^ in Gldc-deficient cells compared to reconstitution of wild-type Gldc (Fig. 6(C) and S4C). These results suggest that polyubiquitination of GLDC^K636^ inhibits CD8^+^ T cell-mediated immunosurveillance. In addition, in line with the reduced tumor burden (Fig. 6(D)), *in vivo* analysis of Gldc^K−R^-reconstituted tumors revealed higher MHC-I levels on tumor cells compared to wild-type Gldc-reconstituted tumors (Fig. 6(E)), accompanied by increased numbers of CD8^+^ T cells in the TME (Fig. 6(F)). The tumor-infiltrating CD8^+^ T cells in Gldc^K−R^-reconstituted tumors had an increased effector functions compared to those from the parental wild-type Gldc-reconstituted tumors, evidenced by their capacities to secrete IFNγ and GzmB (Fig. 6(G)). Collectively, our data suggest that polyubiquitination of GLDC^K636^ inhibits MHC-I expression and CD8^+^ T cell immunity.

### 2.7 Dasatinib improves the antitumor effects of PD-1 blockade therapy

**Fig. 7:**
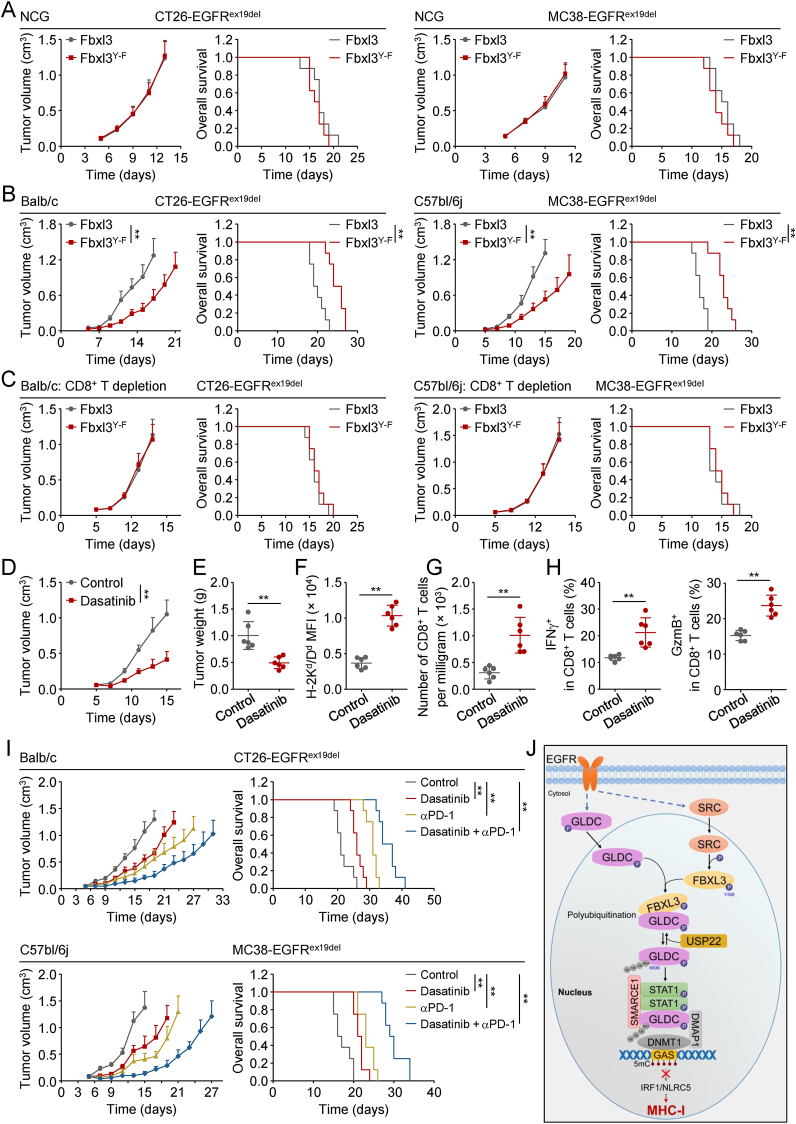
**Dasatinib sensitizes the antitumor effects of PD-1 blockade therapy.** (A–C) Effects of FBXL3 mutation on tumor growth. Fbxl3-deficient (g*Fbxl3*) CT26-EGFR^ex19del^ or MC38-EGFR^ex19del^ cells (5 × 10^5^) reconstituted with mouse wild-type Fbxl3 or Fbxl3^Y306F^ (Fbxl3^Y−F^) were subcutaneously injected into indicated mice. On day 5 after tumor cell inoculation, tumor sizes were measured every two days by caliper. Mice were sacrificed when the tumor size was bigger than 15 mm of the mean tumor diameter, tumor volume exceeded 2000 mm^3^, or tumor had ulcers with diameter reached 10 mm. Graph shows mean ± SEM, *n* = 8 (**A**), *n* = 8 (**B**), *n* = 9 (**C**). Data were analyzed using two-way ANOVA with GraphPad Prism 8. Kaplan–Meier survival curves and corresponding log-rank (Mantel-Cox) tests were used to evaluate the statistical differences between groups in survival studies. ^∗∗^*P* < 0.01. (D and E) Dasatinib inhibits tumor growth. CT26-EGFR^ex19del^ cells (5 × 10^5^) were subcutaneously injected into Balb/c mice. On day 5 after tumor cell inoculation, mice were intraperitoneally injected with Control or Dasatinib (10 mg/(kg·day)). Tumor sizes were measured every two days by caliper from day 5. Tumor-bearing mice were euthanized on day 18, and then tumor tissues were separated from the mice. Tumor weights were measured by Analytical Balance. Graph shows mean ± SEM, *n* = 6. Data were analyzed using a student's unpaired *t*-test with GraphPad Prism 8. ^∗∗^*P* < 0.01. (F) Effects of Dasatinib on MHC-I surface expression of tumor cells *in vivo.* CT26-EGFR^ex19del^ cells (5 × 10^5^) were subcutaneously injected into Balb/c mice. On day 5 after tumor cell inoculation, mice were intraperitoneally injected with Control or Dasatinib (10 mg/(kg·day)). Tumor-bearing mice were euthanized on day 16, and then tumor tissues were separated from the mice. Tumor cells were isolated from the indicated tumor tissues, stained with the indicated antibodies and analyzed by flow cytometry. Graph shows mean ± SEM, *n* = 6 independent samples. Data were analyzed using a student's unpaired *t*-test with GraphPad Prism 8. ^∗∗^*P* < 0.01. (G) Effects of Dasatinib on the numbers of CD8^+^ T cells in TME. TILs were isolated from the CT26-EGFR^ex19del^ tumor tissues in Fig. 7(D). TILs were stained with the indicated antibodies and analyzed by flow cytometry. CD8^+^ T cells were sorted from CD45^+^ populations. Graph shows mean ± SEM, *n* = 6 independent samples. Data were analyzed using a student's unpaired *t*-test with GraphPad Prism 8. ^∗∗^*P* < 0.01. (H) Dasatinib treatment increases tumor infiltrating CD8^+^ cytotoxic T cells. TILs were isolated from the CT26-EGFR^ex19del^ tumor tissues in Fig. 7D. TILs were stained with the indicated antibodies and analyzed by flow cytometry. Graph shows mean ± SEM, *n* = 6 independent samples. Data were analyzed using a student's unpaired *t*-test with GraphPad Prism 8. ^∗∗^*P* < 0.01. (I) Dasatinib treatment sensitized the antitumor effects of PD-1 blocking antibody. CT26-EGFR^ex19del^ or MC38-EGFR^ex19del^ cells (5 × 10^5^) were subcutaneously injected into Balb/c or C57bl/6j mice, respectively. On day 5 after tumor cell inoculation, mice were intraperitoneally injected with Control, Dasatinib (10 mg/(kg·day)) or anti-PD-1 (100 μg per mouse, once every three days). Tumor sizes were measured every two days by caliper from day 5. Mice were sacrificed when the tumor size was bigger than 15 mm of the mean tumor diameter, tumor volume exceeded 2000 mm^3^, or tumor had ulcers with diameter reached 10 mm. Graph shows mean ± SEM, *n* = 8. Data were analyzed using two-way ANOVA with GraphPad Prism 8. Kaplan–Meier survival curves and corresponding log-rank (Mantel-Cox) tests were used to evaluate the statistical differences between groups in survival studies. ^∗∗^*P* < 0.01. (J) A model on the regulatory mechanism of nuclear GLDC inhibition of MHC-I expression.

We next investigated the biological functions of FBXL3^Y306^ phosphorylation. Flow cytometry analysis showed that reconstitution of wild-type FBXL3 but not FBXL3^Y306F^ abrogated the up-regulation of MHC-I in FBXL3-deficient CT26-EGFR^ex19del^ or MC38-EGFR^ex19del^ cells (Fig. S5). Tumor growth and animal survival in NCG mice subcutaneously injected with Fbxl3^Y306F^ (Fbxl3^Y−F^)-reconstituted CT26-EGFR^ex19del^ or MC38-EGFR^ex19del^ cells did not show significant difference in comparison to those injected with wild-type Fbxl3-reconstituted cells (Fig. 7(A)). These results suggest that phosphorylation of Fbxl3^Y306^ has no effect on tumor cell proliferation. Next, we subcutaneously injected the wild-type Fbxl3 or Fbxl3^Y−F^-reconstituted CT26-EGFR^ex19del^ or MC38-EGFR^ex19del^ cells in immune-competent Balb/c or C57bl/6j mice respectively, and found that reconstitution of Fbxl3^Y−F^ in these cells suppressed tumor growth and improved the overall survival compared to reconstitution of wild-type Fbxl3 in these cells (Fig. 7(B)). Depletion of CD8^+^ T cells completely abrogated the tumor-suppressive function induced by reconstitution of Fbxl3^Y−F^ in Fbxl3-deficient cells compared to reconstitution of wild-type Fbxl3 (Fig. 7(C)). These results suggest that phosphorylation of Fbxl3^Y306^ promotes CD8^+^ T cell immunosurveillance.

Considering SRC-mediated phosphorylation of FBXL3 is essential for GLDC-dependent MHC-I suppression. We further tested the anti-tumor effects of SRC inhibitor in mouse tumor model. Dasatinib is an inhibitor of SRC. Administration of Dasatinib alone significantly suppressed tumor growth in mice (Fig. 7(D)). In addition, in line with the reduced tumor burden (Fig. 7(E)), *in vivo* analysis of Dasatinib administration tumors revealed higher MHC-I levels on tumor cells compared to control tumors (Fig. 7(F)), accompanied by increased numbers of CD8^+^ T cells in the TME (Fig. 7(G)). The tumor-infiltrating CD8^+^ T cells in Dasatinib administration tumors had an increased effector functions compared to those from control tumors, evidenced by their capacities to secrete IFNγ and GzmB (Fig. 7(H)). Collectively, our data suggest that Dasatinib inhibits MHC-I expression and CD8^+^ T cell immunity. We further investigated the anti-tumor effects of Dasatinib combined with PD-1 blockade. Consistently, Dasatinib significantly increased the efficacies of PD-1 blocking antibody on tumor suppression and overall survival of mice (Fig. 7(I)). Taken together, these results suggest that administration of SRC inhibitor leads to increased efficacies of ICB therapy.

## 3 Discussion

Posttranslational modifications are known to be critical for regulation of protein functions, such as activity, degradation, localization and conformation (Hu et al., 2016; Liu et al., 2021; Song et al., 2020; Xia et al., 2019). In our previous studies, we found that upon EGFR activation, SRC-mediated phosphorylation of GLDC induces its nuclear translocation. Nuclear GLDC functions as a co-repressor of STAT1, inhibiting STAT1-dependent transcriptional activation by recruiting SMARCE1 and DMAP1, which downregulates the transcription of downstream MHC-I genes, enabling tumor cells to evade CD8^+^ T cell-mediated immunosurveillance. However, it is unclear whether nuclear GLDC is regulated by posttranslational modifications and how its posttranslational modifications contribute to regulation of gene transcription. In this study, we showed that polyubiquitination of GLDC^K636^ in the nucleus drives the binding of GLDC with SMARCE1 and DMAP1. EGFR activation triggers SRC-mediated FBXL3 phosphorylation at Y306, enabling its nuclear interaction with GLDC and subsequent K63-linked polyubiquitination of GLDC. Our findings underscore the essential roles of posttranslational modifications in GLDC-mediated suppression of MHC-I expression and cancer immune evasion.

FBXL3 is a part of the SCF ubiquitin ligase complex (Xing et al., 2013). Previous studies have primarily characterized FBXL3 as a regulator of circadian rhythm and tumor growth (Huber et al., 2016; Parico and Partch, 2020; Xing et al., 2013). Our experimental results demonstrated that FBXL3 is involved in the GLDC-mediated immune escape process. EGFR activation caused K63-linked polyubiquitination of GLDC at K636 in the nucleus. We identified FBXL3 as the E3 ubiquitin ligase responsible for K63-linked polyubiquitination of GLDC at K636. FBXL3 was associated with GLDC in the nucleus upon EGFR activation. Overexpression of FBXL3 but not its inactive mutants promoted K63-linked polyubiquitination of GLDC following EGF stimulation, whereas FBXL3-deficiency impaired EGF-induced K63-linked polyubiquitination of GLDC. Mutation of K636 of GLDC to arginine impaired its K63-linked polyubiquitination induced by EGF stimulation, and overexpression of FBXL3 failed to catalyze K63-linked polyubiquitination of GLDC^K636R^.

Our experiments further investigated that FBXL3 is phosphorylated by SRC at Y306 upon EGFR activation, and this phosphorylation of FBXL3 induces its interaction with GLDC in the nucleus. Mutation of Y306 of FBXL3 to phenylalanine impaired EGF-induced tyrosine phosphorylation of FBXL3. Inhibition of SRC or SRC-deficiency prevented EGF-induced tyrosine phosphorylation of FBXL3^Y306^. GLDC was associated with wild-type FBXL3 but not FBXL3^Y306F^ following EGF treatment or overexpression of SRC. FBXL3^Y306F^ failed to promote K63-linked polyubiquitination of GLDC following EGF stimulation. EGF stimulation increased K63-linked polyubiquitination of GLDC in wild-type FBXL3-reconstituted FBXL3-deficient cells but not in FBXL3^Y306F^-reconstituted FBXL3-deficient cells. Y306 phosphorylation of FBXL3 is essential for mediating GLDC polyubiquitination and the interaction of FBXL3 with GLDC. This phosphorylation may induce a conformational change in FBXL3 that enhances its substrate recognition or binding affinity for GLDC.

Our experiments also suggest that USP22 deubiquitinates GLDC. Overexpression of USP22 but not its inactive mutants removed K63-linked polyubiquitin moieties from GLDC induced by EGF stimulation or overexpression of SRC. Knockout of USP22 increased EGF-induced K63-linked polyubiquitination of GLDC. Moreover, USP22 removed K63-linked polyubiquitination moieties of GLDC conjugated by FBXL3.

Multiple lines of evidence indicate that the K636 polyubiquitination of GLDC is essential for GLDC mediated suppression of MHC-I expression. Reconstitution of wild-type GLDC but not GLDC^K636R^ abrogated the up-regulation of MHC-I in GLDC-deficient cells. FBXL3-deficiency increased MHC-I surface expression. USP22-deficiency impaired the expression of MHC-I. In addition, FBXL3 and USP22 failed to regulate the expression of MHC-I in GLDC-deficient cells. Reconstitution of wild-type FBXL3 but not FBXL3^Y306F^ inhibited the expression of MHC-I in FBXL3-deficient cells.

Our previous studies have demonstrated that nuclear GLDC hijacks the STAT1 co-activator SMARCE1 to inhibit the binding of STAT1 to promoter regions of *IRF1* and *NLRC5* genes, and the GLDC/SMARCE1/STAT1 complex also recruits DMAP1/DNMT1 to induce promoter DNA hypermethylation of *IRF1* and *NLRC5* genes, resulting in transcriptional inhibition of the downstream MHC-I genes. Our experiments further indicated that polyubiquitination of GLDC at K636 determines the binding of GLDC with SMARCE1 and DMAP1. Reconstitution of wild-type GLDC but not GLDC^K636R^ abrogated up-regulation of the mRNA levels of *HLA-A*, *HLA-B*, *HLA-C*, *B2M, IRF1* and *NLRC5* genes, up-regulation of the binding of STAT1 and SMARCE1 to *IRF1* promoter region, down-regulation of DNMT1 binding to *IRF1* promote region, and down-regulation of the levels of *IRF1* promoter DNA methylation in GLDC deficient cells. GLDC^K636R^ failed to interact with SMARCE1 or DMAP1 following EGF stimulation. FBXL3-deficiency abolished the interaction of GLDC with SMARCE1 or DMAP1. Reconstitution of wild-type FBXL3 but not FBXL3^Y306F^ in FBXL3-deficient cells restored the interaction of SMARCE1 or DMAP1 with GLDC.

Based on our results, we propose a mechanistic model whereby nuclear GLDC inhibits MHC-I expression: hyperactive EGFR signaling in cancerous cells activates SRC, which mediates phosphorylation of FBXL3 at Y306, enabling its interaction with nuclear GLDC. This interaction promotes K63-linked polyubiquitination of GLDC at K636, facilitating its recruitment of SMARCE1 and DMAP1 to inhibit STAT1-triggered transcriptional activation, resulting in transcriptional inhibition of downstream MHC-I genes (Fig. 7(J)). Consistent with this model, our experiments indicated that polyubiquitination of GLDC^K636^ and phosphorylation of FBXL3^Y306^ down-regulated MHC-I levels in tumor cells and inhibited CD8^+^ T cell immunity in TME. In addition, inhibitors of SRC (Dasatinib) treatment up-regulated MHC-I levels in tumor cells, increased numbers of CD8^+^ T cells in tumors, improved tumor-specific CD8^+^ T cells functions in TME and sensitized antitumor effects of anti-PD-1 therapy. In conclusion, our findings reveal that phosphorylation of FBXL3 targets nuclear GLDC polyubiquitination to drive GLDC-triggered transcriptional inhibition of MHC-I genes, resulting in promoting EGFR-activated tumor cells to evade CD8^+^ T cell-mediated immunosurveillance. As validated in our study, regulation of these mechanisms would provide new targets for the development of ICB-based combination immunotherapy strategies.

## 4 Materials and methods

### 4.1 Reagents and antibodies

Details regarding the commercially available reagents utilized in this investigation are provided in Table S2. Information on the commercially available antibodies employed is presented in Table S3. The antibody specifically recognizing phosphorylated Y306 on FBXL3 was generated by immunizing rabbits with a synthetic peptide corresponding to human FBXL3 (_301_DPFFR(Y-p)EIPATH_312_) by ABclonal Technology (Wuhan).

### 4.2 Cells

A549 and H1299 cell lines were procured from the American Type Culture Collection. The CT26 and MC38 cell lines were provided by Dr. Jinfang Zhang of Wuhan University. HEK293 cells were a gift from Dr. Gary Johnson (National Jewish Health, Denver, CO). All cell lines were maintained in DMEM (GIBCO, Catalog #C11995500) supplemented with 10% FBS (Cell Max, Catalog #SA211.02) and 1% penicillin-streptomycin (GIBCO, Catalog #15140-122). All cultures were confirmed to be mycoplasma-free.

### 4.3 Constructs

Mammalian expression plasmids for Flag-, HA-, or Myc-tagged GLDC, SRC, FBXL3, FBXL11, NF-X1, TRAF4, RNF138, USP5, USP10, USP11, USP22, UCHL1, UCHL5, OTUB1, BRCC3 and their mutants were constructed by standard molecular biology techniques. Guide-RNA plasmids targeting GLDC, FBXL3, USP22 and SRC were constructed into a Lenti-CRISPR-V2 vector, which was provided by Dr. Shu-Wen Wu (Wuhan University).

### 4.4 Transfection

Transfection of HEK293 cells was carried out via the standard calcium phosphate precipitation method. To ensure equal DNA amounts across all transfections, an empty control plasmid was added to each reaction.

### 4.5 Flow cytometry analysis

For analysis, cells were stained with the indicated antibodies for 45 min at 4 °C. Data acquisition was performed on a BD Fortessa X-20 instrument using FACSDiva 7 software, following an exemplified gating strategy. Subsequent data processing was conducted with FlowJo software. A list of the antibodies used for flow cytometric analyses in this study can be found in Table S4.

### 4.6 CRISPR-Cas9 knockout

Double-stranded oligonucleotides corresponding to the target sequences were cloned into the Lenti-CRISPR-V2 vector. These vectors were then co-transfected with packaging plasmids into HEK293 cells. The resulting viral supernatants were harvested two days post-transfection, filtered through a 0.45 μm membrane (Millipore), and used to infect target cells in the presence of polybrene (8 μg/mL). Infected cells were subjected to selection with puromycin (A549: 2 μg/mL, H1299: 2 μg/mL, CT26: 4 μg/mL, MC38: 3 μg/mL) for a minimum of six days. The sequences of the gRNAs used are detailed in Table S5.

### 4.7 Mouse tumor models

Mice were housed five per cage under a 12-h light/dark cycle in a temperature-controlled room (23-25 °C) with a relative humidity of 40%-70%, with ad libitum access to food and water. All animals were allowed a minimum of seven days for acclimation prior to experimentation. All animal procedures were conducted in compliance with the Institutional Animal Care and Use Committee guidelines and received approval from the Animal Care and Ethics Committee of Wuhan University Medical Research Institute. Age- and sex-matched Balb/c, C57bl/6j, or NCG mice (all 6-8 weeks of age) were anesthetized and subcutaneously injected with the specified mouse tumor cells (5 × 10^5^ cells in 200 μL PBS). Mice were euthanized when the mean tumor diameter exceeded 15 mm, the tumor volume reached 2000 mm^3^, or when they met other humane endpoint criteria.

### 4.8 Animal survival studies

For survival assessments, mice were sacrificed when the mean tumor diameter was greater than 15 mm, the tumor volume surpassed 2000 mm^3^, or if the tumor developed ulcers with a diameter of 10 mm. Statistical analysis was performed using GraphPad Prism 8 software. Kaplan–Meier survival curves and the corresponding log-rank (Mantel-Cox) tests were utilized to evaluate statistical differences between groups, with a *P*-value of less than 0.05 considered significant.

### 4.9 *In vivo* mouse CD8^+^ T cells depletion experiments

To deplete CD8^+^ T cells in Balb/c and C57bl/6j mice, anti-CD8α antibodies (A2102, dissolved in PBS, Selleck) were administered via intraperitoneal injection every three days, starting three days prior to tumor cell inoculation. A total of five injections were given: an initial dose of 200 μg per mouse, followed by four doses of 100 μg per mouse. The efficacy of depletion was confirmed by flow cytometry.

### 4.10 *In vivo* experimental therapy in syngeneic mouse tumor models

For Dasatinib therapy in Balb/c mice, either a control or Dasatinib (D125110, 10 mg/kg/day, Aladdin) was administered via intraperitoneal injection five days after tumor cell inoculation. Tumor dimensions were measured with calipers every two days. Tumor-bearing mice were euthanized on day 18, and tumor-infiltrating lymphocytes (TILs) were analyzed by flow cytometry.

For combined Dasatinib and anti-PD-1 therapy in Balb/c and C57bl/6j mice, treatment with a control, Dasatinib (10 mg/kg/day), or anti-PD-1 (100 μg per mouse, once every three days) was initiated five days after inoculation with CT26-EGFR^ex19del^ (Balb/c) or MC38-EGFR^ex19del^ (C57bl/6j) cells. Tumor size and mouse survival were monitored every two days starting from day five.

### 4.11 Isolation of tumor infiltrated immune cells

Tumor tissues were excised from mice and minced. The tissue fragments were suspended in 2 mL of tumor digestion buffer (1 × HBSS containing 5 mg/mL collagenase II and 0.1% DNase I) and rotated at 37 °C for 1 h. The resulting cell suspension was filtered through a 70-μm filter to obtain a single-cell suspension. Lymphocytes were then isolated using density-gradient centrifugation with 40% and 70% Percoll (GE). The TILs were stained with fluorescently labeled antibodies for various markers. Data acquisition and analysis were performed on a BD Fortessa X-20 with FACSDiva 7 software, following a standard gating strategy, and processed with FlowJo software.

### 4.12 Co-immunoprecipitation and immunoblotting analysis

Cells were lysed in 1 mL of NP-40 lysis buffer (20 mM Tris-HCl, 150 mM NaCl, 1 mM EDTA, 1% Nonidet P-40, 1% Triton X-100, 10 μg/mL aprotinin, 10 μg/mL leupeptin, and 1 mM PMSF). For each immunoprecipitation, a 0.4 mL aliquot of lysate was incubated with 0.5μg-2 μg of the specified antibody or control IgG and 35 μL of a 1:1 slurry of Protein-G Sepharose at 4 °C for 3 h. The Sepharose beads were washed three times with 1 mL of lysis buffer containing 500 mM NaCl. The precipitated proteins were resolved by SDS-PAGE and subjected to immunoblotting analysis with the indicated antibodies.

### 4.13 Cell fractionation assays

Cells were either left untreated or stimulated with EGF for specified durations. Nuclear and cytoplasmic fractions were separated using the NE-PER Nuclear and Cytoplasmic Extraction Reagents (Thermo, Catalog #78835).

### 4.14 Quantitative real-time PCR (qPCR)

Total RNA was isolated for qPCR analysis to quantify the mRNA levels of the genes of interest. The data presented represent the relative abundance of target mRNAs, normalized to the levels of GAPDH or β-actin. qPCR data were collected on a Bio-Rad CFX96 (Version 3.1) and analyzed with Bio-Rad CFX Manager (Version 3.1). The sequences of the gene-specific primers are listed in Table S6.

### 4.15 Recombinant protein purification

To produce Flag-FBXL3 and Flag-FBXL3^Y306F^, their respective mammalian expression plasmids were transfected into HEK293 cells. Cells were lysed 24 h post-transfection. Flag antibody-conjugated beads were used for immunoprecipitation over 4 h at 4 °C. After washing the beads three times with lysis buffer, the bound proteins were eluted with 3 × Flag peptides in 250 mM Tris-HCl, pH 8.0. The purified Flag-FBXL3 and Flag-FBXL3Y306F were then used for *in vitro* kinase assays.

### 4.16 *In vitro* kinase assay

The HEK293-purified Flag-FBXL3 and Flag-FBXL3Y306F were incubated with commercial active GST-SRC in kinase buffer (60 mM HEPES pH 7.5, 5 mM MgCl_2_, 5 mM MnCl_2_, 3 μM Na_3_VO_4_, 1.25 mM DTT and 20 μM ATP). The 50 μL reactions were incubated at 37 °C for 30 min. The reactions were terminated by adding SDS-PAGE loading buffer and heating at 95 °C for 5 min. The proteins were then analyzed by immunoblotting with the indicated antibodies.

### 4.17 Chromatin immunoprecipitation (ChIP)

The ChIP assay was performed according to the manufacturer's instructions. Ten million cells were cross-linked with 1% formaldehyde for 10 min, quenched with 0.125 M glycine for 5 min at 37 °C, and lysed in SDS Lysis Buffer. Chromatin was sheared by sonication using a Bioruptor Pico Sonifier to yield DNA fragments of 200-1000 bp. The lysate was diluted 10-fold in ChIP Dilution Buffer and precleared with 60 mL agarose beads for 30 min. The supernatant was incubated with 2 μg of the specified antibodies overnight at 4 °C. Antibody-chromatin complexes were captured with protein A agarose/salmon sperm DNA beads (Sigma, Catalog #16-157) for 1 h at 4 °C. Following reverse cross-linking, the DNA was subjected to qPCR analysis using the primers listed in Table S7 (Liu et al., 2023).

### 4.18 Methylated DNA immunoprecipitation (MeDIP)

The MeDIP procedure was conducted as previously described (Thu et al., 2009). Genomic DNA was extracted using the QIAamp DNA Mini Kit (Qiagen, Cat. #51304) as per the manufacturer's protocol. Two micrograms of genomic DNA were sheared by sonication (Bioruptor Pico Sonifier) to fragments of 200bp-500 bp for the MeDIP assay. The assay employed a monoclonal antibody against 5-mC (Abcam, clone: 33D3, Cat. #ab10805) coupled to magnetic Dynabeads anti-mouse IgG (Invitrogen, Cat. #M-280). The sheared DNA was incubated with the antibody-bead complex overnight at 4 °C. After five washes with immunoprecipitation wash buffer, the DNA was eluted from the beads in digestion buffer [10 mmol/L EDTA, 10 mmol/L Tris-HCl (pH 8.0), 0.5% SDS, 50 mmol/L NaCl] with proteinase K at 50 °C for 30 min. Five percent of the input DNA was used as a control. The primers used for qPCR are listed in Table S7.

### 4.19 Mass spectrometry

HEK293/EGFR cells (1 × 10^8^) were transfected with Flag-tagged human GLDC for 24 h and subsequently treated with or without EGF (100 ng/mL) for 6 h. Flag-GLDC was immunoprecipitated with an anti-Flag antibody, desalted, and then analyzed by mass spectrometry. The mass spectrometry analysis was performed by SpecAlly (Wuhan) Life Science and Technology Company, following previously described methods (Liu et al., 2021, 2023).

### 4.20 Statistics and reproducibility

Statistical analysis was performed using a student's unpaired *t*-test, multiple t-tests, or two-way ANOVA with GraphPad Prism 8. Correlation studies were analyzed using the Spearman rank correlation test. The number of asterisks indicates the degree of significance relative to the *P*-value, which is provided in each figure legend. All biochemical experiments, particularly immunoblotting, were repeated at least twice with consistent results. The reproducibility of other experiments is detailed in the respective figure legends.

## CRediT authorship contribution statement

**Rui Liu:** Writing – review & editing, Writing – original draft, Visualization, Validation, Supervision, Software, Resources, Project administration, Methodology, Investigation, Funding acquisition, Formal analysis, Data curation, Conceptualization. **Shu Li:** Writing – review & editing, Visualization, Validation, Supervision, Resources, Project administration, Investigation, Funding acquisition, Formal analysis, Conceptualization.

## Declaration of competing interest

The authors declare that they have no known competing financial interests or personal relationships that could have appeared to influence the work reported in this paper.

## Data Availability

All the data supporting the findings of this study are available within the article and its supplementary information files, or can be obtained from the corresponding author upon reasonable request.
