# Supplementary material for: Phosphorylation of FBXL3 mediates GLDC polyubiquitination to suppress MHC-I expression and promote cancer immune evasion
Source: Cell Insight. 2026 Feb 3;5(2):100308. doi: 10.1016/j.cellin.2026.100308 (PMC12924200; doi:10.1016/j.cellin.2026.100308)
Supplement: Multimedia component 1 [file mmc1.docx]

| **Table S1. A list of deubiquitinate enzymes in mass spectrometry analysis.** | | | |  |
| --- | --- | --- | --- | --- |
| **Accession** | **Gene** | **Peptides** | **Intensity** | |
| P45974 | USP5 | 2 | 31154 | |
| Q14694 | USP10 | 5 | 189830 | |
| P51784 | USP11 | 1 | 10737 | |
| Q9Y4E8 | USP15 | 8 | 311690 | |
| Q9UPT9 | USP22 | 3 | 191790 | |
| P09936 | UCHL1 | 2 | 31495 | |
| Q9Y5K5 | UCHL5 | 3 | 62068 | |
| Q96FW1 | OTUB1 | 1 | 134330 | |
| P46736 | BRCC3 | 1 | 17297 | |

| **Table S2. A list of reagents used in the study** | | |
| --- | --- | --- |
| **Reagent** | **Supplier** | **Catalog No.** |
| Human EGF | Peprotech | #100-15 |
| Human IFNγ | Peprotech | #300-02 |
| Mouse Ifnγ | Peprotech | #3315-05 |
| Afatinib | Aladdin | #401422 |
| Saracatinib | Aladdin | #S125141 |
| Ruxolitinib | Aladdin | #R126338 |
| Amuvatinib | Aladdin | #M127412 |
| Recombinant human GST-SRC protein | SinoBiological | #S19-18G |
| Polybrene | Millipore | #3924803 |
| Hygromycin | InvivoGen | #ant-hg-1 |
| Blasticidin | InvivoGen | #ant-bl-05 |
| SYBR | Bio-Rad | #1725125 |
| Cycloheximide | Sigma | #239763 |

| **Table S3. A list of primary antibodies used in the study.** | | | | |
| --- | --- | --- | --- | --- |
| **Antibody** | **Supplier** | **Catalog No.** | **Appl.^a^** | **Usage** |
| Mouse anti-Flag M2 antibody clone M2 | Sigma-Aldrich | #F3165 | WB/IP | 1:2000/1 μg |
| Mouse anti-HA. 11 Epitope Tag antibody clone 16B12 | BioLegend | #901515 | WB/IP | 1:2000/1 μg |
| Mouse anti-Myc-Tag antibody clone 9B11 | Cell Signaling Technology | #2276S | WB | 1:2000 |
| Mouse anti-β-actin antibody clone AC-74 | Sigma-Aldrich | #A2228 | WB | 1:5000 |
| Rabbit anti-GLDC polyclonal antibody | NOVUS | #NBP1-32907 | WB/IP/IF | 1:1000/1 μg  /1:200 |
| Rabbit anti-GLDC polyclonal antibody | GeneTex | #GTX110267 | WB/IP | 1:1000/1 μg |
| Mouse anti-β-tubulin polyclonal antibody | ABclonal | #AC021 | WB | 1:5000 |
| Rabbit anti-Lamin B1 polyclonal antibody | Proteintech | #12987-1-AP | WB | 1:7500 |
| Rabbit anti-USP22 antibody clone EPR18945 | Abcam | #ab195289 | WB | 1:1000 |
| Rabbit anti-FBXL3 polyclonal antibody | GeneTex | #GTX110755 | WB/IP | 1:1000/1 μg |
| Rabbit anti-pSRC (Tyr419) polyclonal antibody | Abcam | #ab4816 | WB | 1:1000 |
| Rabbit anti-SRC antibody clone 36D10 | Cell Signaling Technology | #2109 | WB | 1:1000 |
| Rabbit anti-SMARCE1 polyclonal antibody | FORTIS | #A300-810A | WB/IP  /ChIP | 1:1500/2 μg  /2 μg |
| Rabbit anti-DMAP1 polyclonal antibody | Proteintech | #10411-1-AP | WB/IP | 1:1500/2 μg |
| Rabbit anti-DNMT1 antibody clone D63A6 | Cell Signaling Technology | #5032 | WB | 1:1000 |
| Mouse anti-5mC antibody clone 33D3 | Abcam | #ab10805 | MeDIP | 1 μg |
| Mouse anti-p-Tyr antibody clone P-Tyr-100 | Cell Signaling Technology | #9411 | WB | 1:2000 |
| Rabbit anti-p-Tyr antibody clone P-Tyr-1000 | Cell Signaling Technology | #8954 | WB | 1:2000 |

| **Table S4. A list of flow antibodies used in the study.** | | | |
| --- | --- | --- | --- |
| **Antibody** | **Clone** | **Supplier** | **Catalog No.** |
| FITC anti-human HLA-ABC antibody | W6/32 | BioLegend | #311404 |
| APC anti-human HLA-ABC antibody | W6/32 | BioLegend | #311410 |
| APC anti-mouse H-2K^d/^D^d^ antibody | 34-1-2S | BioLegend | #114714 |
| APC anti-mouse H-2K^b/^D^b^ antibody | 28-8-6 | BioLegend | #114614 |
| FITC anti-mouse CD45.2 antibody | 104 | BioLegend | 109806 |
| PE-Cy7 anti-mouse CD3 antibody | 145-2C11 | BD Biosciences | #552774 |
| PB anti-mouse CD8 antibody | 53-6.7 | BD Biosciences | #558106 |
| PerCP-710 anti-mouse GzmB antibody | NGZB | eBioscience | #46-8898-82 |
| APC anti-mouse IFNγ antibody | XMG1.2 | eBioscience | #17-7311-82 |

| **Table S5. A list of gRNA sequences.** | |
| --- | --- |
| Human *GLDC* | 5’-CAGATCTGGAGATCGTATAT-3’ |
| Human *SRC* #1 | 5’-GGCTTGCTGGGGGTCTGCGAGG-3’ |
| Human *SRC* #2 | 5’-AGCGCCGTGCACGTTCTCGG-3’ |
| Human *FBXL3* #1 | 5’-CTTCACAAGTTTGCCGCAAC-3’ |
| Human FBXL3 #2 | 5’-GGTACGCGCCGGCATCTCGG-3’ |
| Mouse *Fbxl3* | 5’-TCCGCCCGCATCCGAGACGC-3’ |
| Mouse *Gldc* | 5’-TCCCGGAGACACATCGGCCC-3’ |

| **Table S6. A list of qPCR sequences.** | |
| --- | --- |
| Human *GAPDH* | GTCTCCTCTGACTTCAACAGCG |
|  | ACCACCCTGTTGCTGTAGCCAA |
| Human *β-actin* | GCACAGAGCCTCGCCTT |
|  | CCTTGCACATGCCGGAG |
| Human *HLA-A* | AAAAGGAGGGAGTTACACTCAGG |
|  | GCTGTGAGGGACACATCAGAG |
| Human *HLA-B* | CAGTTCGTGAGGTTCGACAG |
|  | CAGCCGTACATGCTCTGGA |
| Human *HLA-C* | CACACCTCTCCTTTGTGACTTCAA |
|  | CCACCTCCTCACATTATGCTAACA |
| Human *B2M* | GAGGCTATCCAGCGTACTCCA |
|  | CGGCAGGCATACTCATCTTTT |
| Human *IRF1* | GAGGAGGTGAAAGACCAGAGCA |
|  | TAGCATCTCGGCTGGACTTCGA |
| Human *NLRC5* | AGTGGCTCTTCCGCTTGGACAT |
|  | CGGAACCCTAAGAACTTGGCTG |

| **Table S7. A list of qPCR sequences for CHIP/MeDIP analysis.** | |
| --- | --- |
| Human *IRF1 #1* | TACTTCCCCTTCGCCGCTA |
|  | CGTCTTGCCTCGACTAAGGAG |
| Human *IRF1 #2* | CCCTGTACTTCCCCTTCGCC |
|  | GCCACCGAGCAATCCAAACA |
| Mouse *Irf1 #1* | TCGCCGCTTAGCTCTACAAC |
|  | TGAAAGCACGTCCTACCTCG |
| Mouse *Irf1 #2* | TGCCTTGTACTTCCCCTTCG |
|  | GGCGCCGCGAAGAAATCTAA |
