# Supplementary material for: Phosphorylation of FBXL3 mediates GLDC polyubiquitination to suppress MHC-I expression and promote cancer immune evasion
Source: Cell Insight. 2026 Feb 3;5(2):100308. doi: 10.1016/j.cellin.2026.100308 (PMC12924200; doi:10.1016/j.cellin.2026.100308)
Supplement: Multimedia component 2 [file mmc2.pptx]

## Slide 1
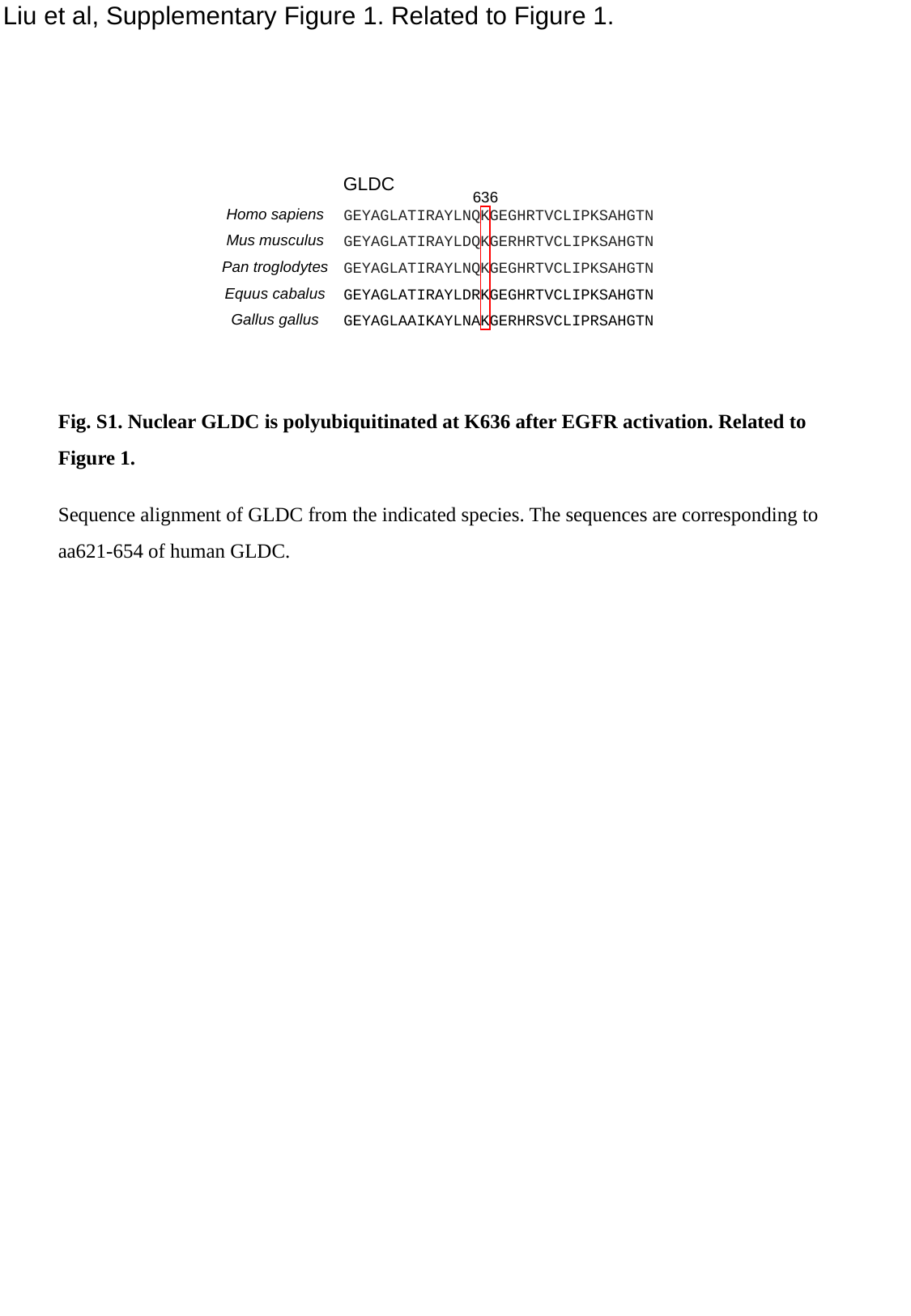

Liu et al, Supplementary Figure 1. Related to Figure 1.
GLDC
636
GEYAGLATIRAYLNQKGEGHRTVCLIPKSAHGTN
GEYAGLATIRAYLDQKGERHRTVCLIPKSAHGTN
GEYAGLATIRAYLNQKGEGHRTVCLIPKSAHGTN
GEYAGLATIRAYLDRKGEGHRTVCLIPKSAHGTN
GEYAGLAAIKAYLNAKGERHRSVCLIPRSAHGTN
Homo sapiens
Mus musculus
Pan troglodytes
Equus cabalus
Gallus gallus
Fig. S1. Nuclear GLDC is polyubiquitinated at K636 after EGFR activation. Related to Figure 1.
Sequence alignment of GLDC from the indicated species. The sequences are corresponding to aa621-654 of human GLDC.

## Slide 2
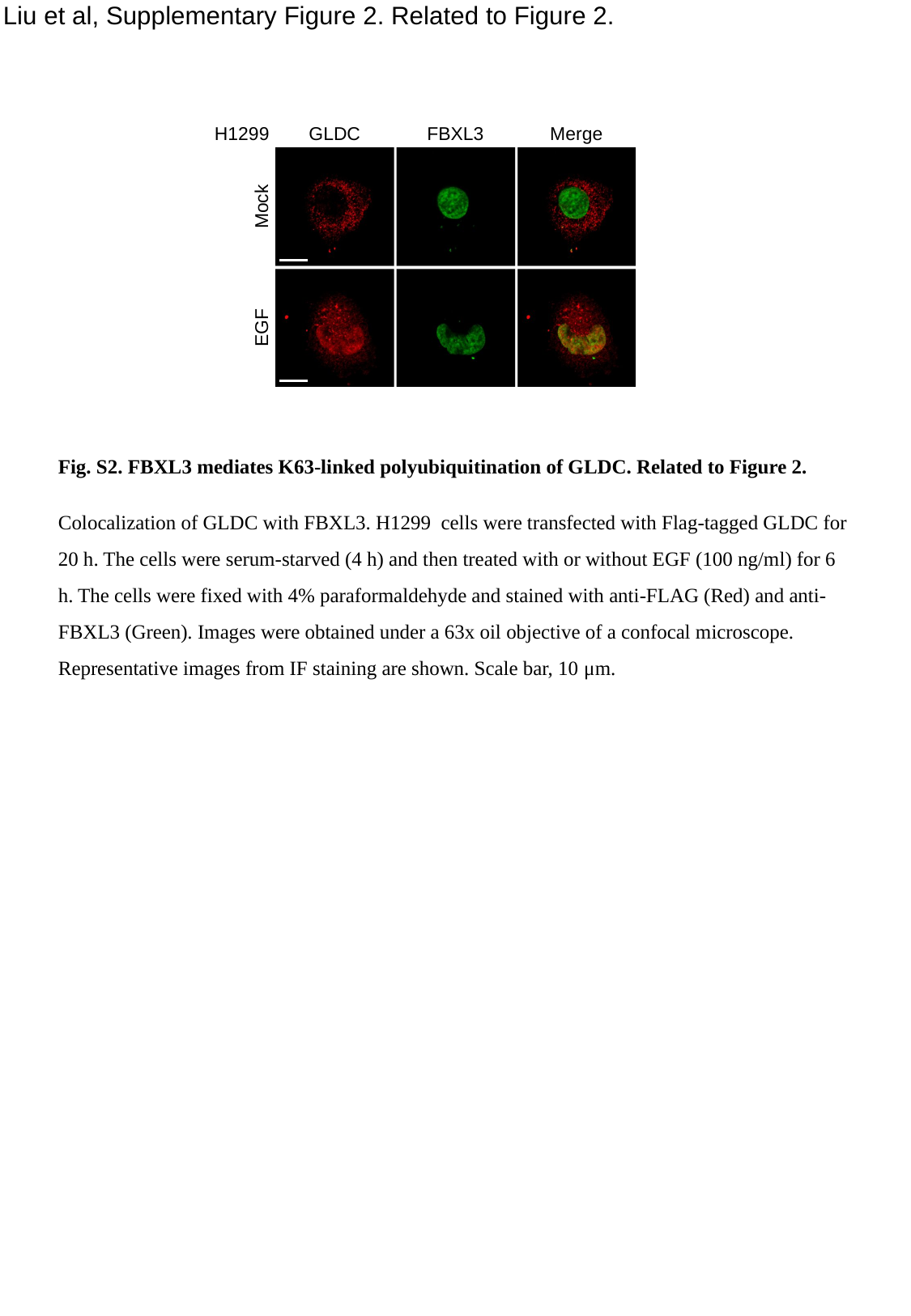

Liu et al, Supplementary Figure 2. Related to Figure 2.
H1299
GLDC
FBXL3
Merge
Mock
EGF
Fig. S2. FBXL3 mediates K63-linked polyubiquitination of GLDC. Related to Figure 2.
Colocalization of GLDC with FBXL3. H1299 cells were transfected with Flag-tagged GLDC for 20 h. The cells were serum-starved (4 h) and then treated with or without EGF (100 ng/ml) for 6 h. The cells were fixed with 4% paraformaldehyde and stained with anti-FLAG (Red) and anti-FBXL3 (Green). Images were obtained under a 63x oil objective of a confocal microscope. Representative images from IF staining are shown. Scale bar, 10 μm.

## Slide 3
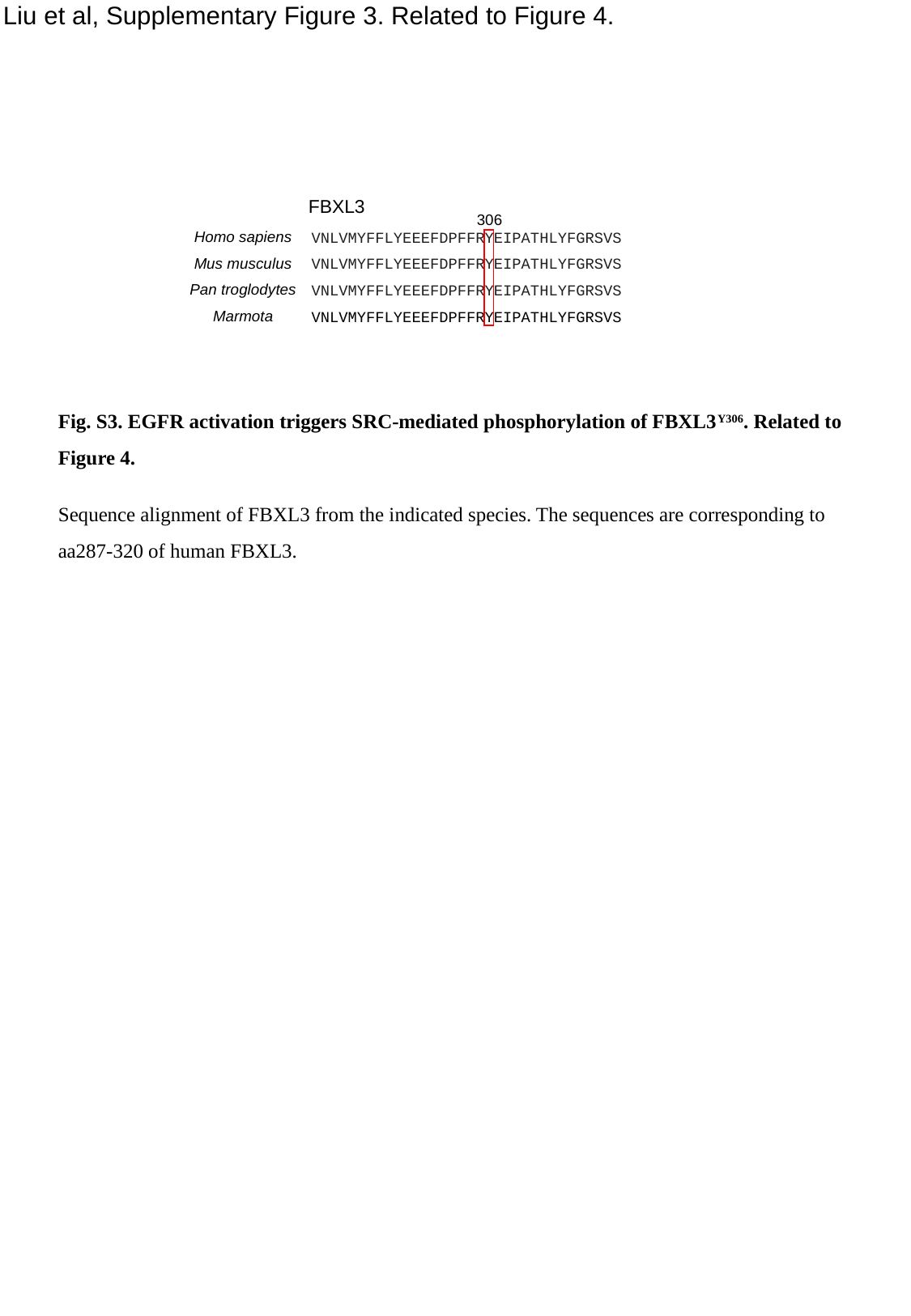

Liu et al, Supplementary Figure 3. Related to Figure 4.
FBXL3
306
VNLVMYFFLYEEEFDPFFRYEIPATHLYFGRSVS
VNLVMYFFLYEEEFDPFFRYEIPATHLYFGRSVS
VNLVMYFFLYEEEFDPFFRYEIPATHLYFGRSVS
VNLVMYFFLYEEEFDPFFRYEIPATHLYFGRSVS
Homo sapiens
Mus musculus
Pan troglodytes
Marmota
Fig. S3. EGFR activation triggers SRC-mediated phosphorylation of FBXL3Y306. Related to Figure 4.
Sequence alignment of FBXL3 from the indicated species. The sequences are corresponding to aa287-320 of human FBXL3.

## Slide 4
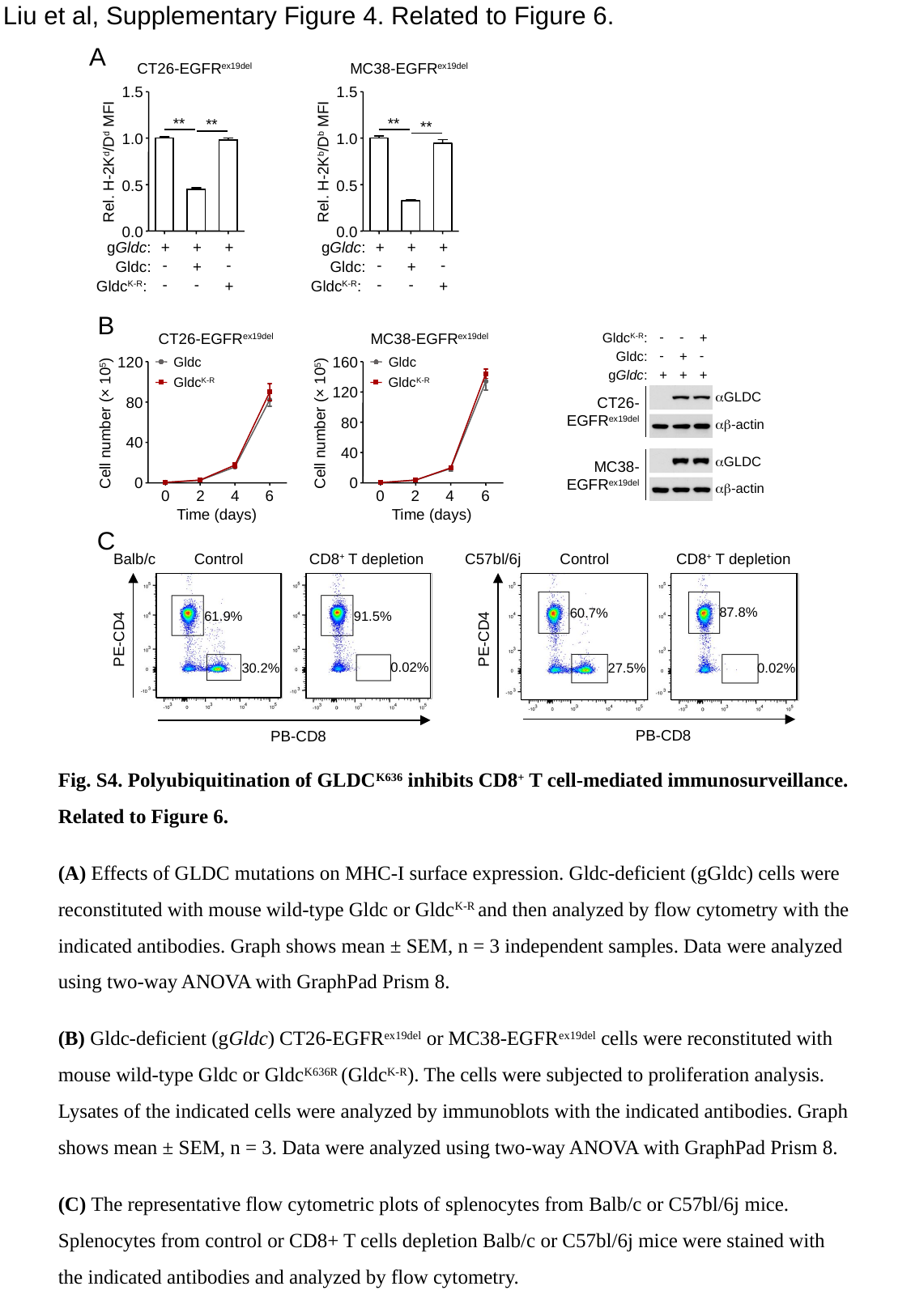

Liu et al, Supplementary Figure 4. Related to Figure 6.
A
CT26-EGFRex19del
MC38-EGFRex19del
1.5
1.5
**
**
**
**
1.0
1.0
Rel. H-2Kd/Dd MFI
Rel. H-2Kb/Db MFI
0.5
0.5
0.0
0.0
gGldc:
+
+
+
gGldc:
+
+
+
-
-
-
-
+
+
Gldc:
Gldc:
-
-
-
-
GldcK-R:
+
GldcK-R:
+
B
-
-
GldcK-R:
+
-
-
Gldc:
+
gGldc:
+
+
+
aGLDC
CT26-
EGFRex19del
ab-actin
aGLDC
MC38-
EGFRex19del
ab-actin
CT26-EGFRex19del
Gldc
GldcK-R
120
80
Cell number (× 105)
40
0
0
2
4
6
Time (days)
MC38-EGFRex19del
160
120
80
Cell number (× 105)
40
0
0
2
4
6
Time (days)
Gldc
GldcK-R
C
Balb/c
Control
CD8+ T depletion
61.9%
91.5%
PE-CD4
0.02%
30.2%
PB-CD8
C57bl/6j
Control
CD8+ T depletion
87.8%
60.7%
PE-CD4
0.02%
27.5%
PB-CD8
Fig. S4. Polyubiquitination of GLDCK636 inhibits CD8+ T cell-mediated immunosurveillance. Related to Figure 6.
(A) Effects of GLDC mutations on MHC-I surface expression. Gldc-deficient (gGldc) cells were reconstituted with mouse wild-type Gldc or GldcK-R and then analyzed by flow cytometry with the indicated antibodies. Graph shows mean ± SEM, n = 3 independent samples. Data were analyzed using two-way ANOVA with GraphPad Prism 8.
(B) Gldc-deficient (gGldc) CT26-EGFRex19del or MC38-EGFRex19del cells were reconstituted with mouse wild-type Gldc or GldcK636R (GldcK-R). The cells were subjected to proliferation analysis. Lysates of the indicated cells were analyzed by immunoblots with the indicated antibodies. Graph shows mean ± SEM, n = 3. Data were analyzed using two-way ANOVA with GraphPad Prism 8.
(C) The representative flow cytometric plots of splenocytes from Balb/c or C57bl/6j mice. Splenocytes from control or CD8+ T cells depletion Balb/c or C57bl/6j mice were stained with the indicated antibodies and analyzed by flow cytometry.

## Slide 5
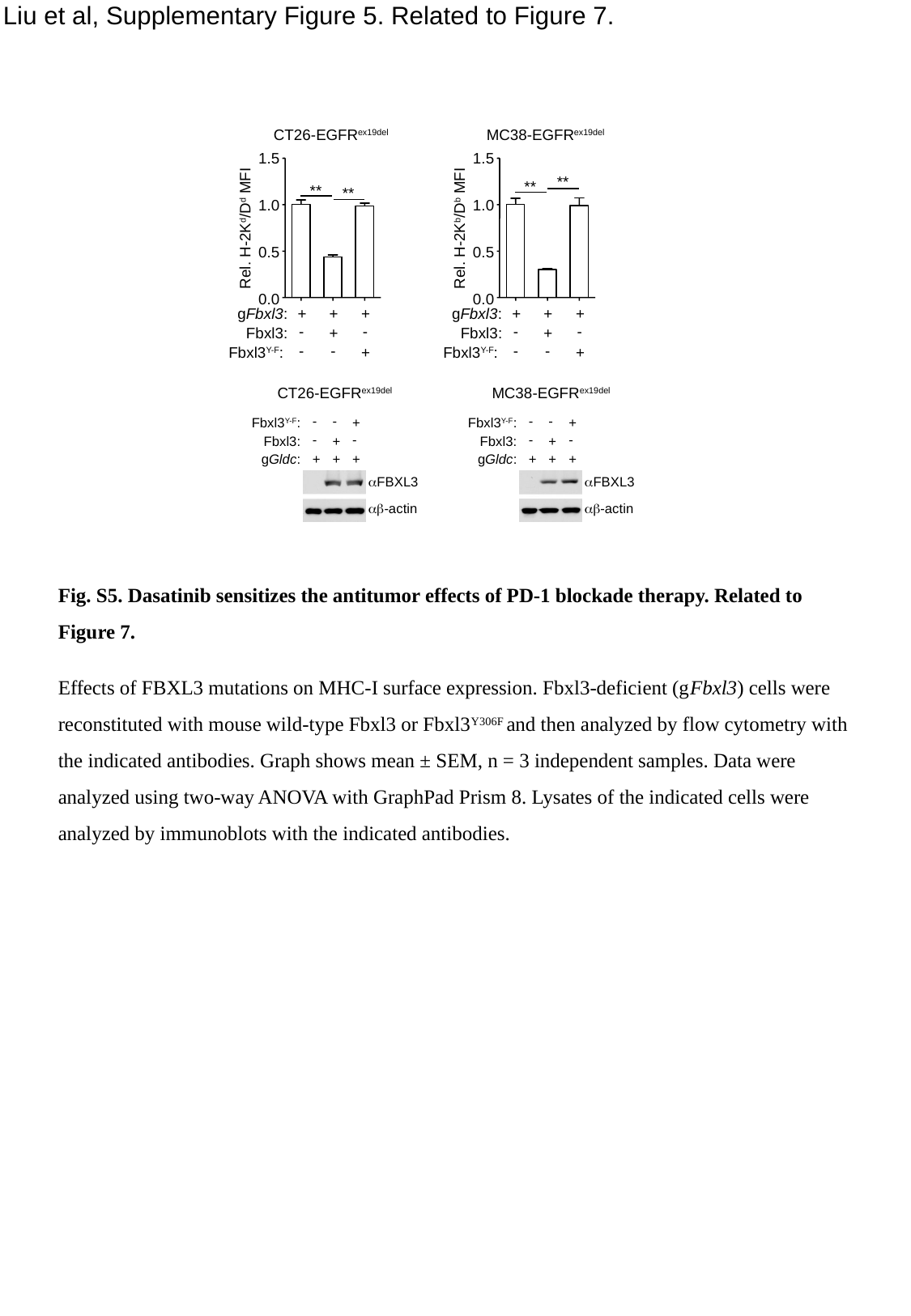

Liu et al, Supplementary Figure 5. Related to Figure 7.
CT26-EGFRex19del
1.5
**
**
1.0
Rel. H-2Kd/Dd MFI
0.5
0.0
gFbxl3:
+
+
+
-
-
+
Fbxl3:
-
-
Fbxl3Y-F:
+
MC38-EGFRex19del
1.5
**
**
1.0
Rel. H-2Kb/Db MFI
0.5
0.0
gFbxl3:
+
+
+
-
-
+
Fbxl3:
-
-
Fbxl3Y-F:
+
CT26-EGFRex19del
-
-
Fbxl3Y-F:
+
-
-
Fbxl3:
+
gGldc:
+
+
+
aFBXL3
ab-actin
MC38-EGFRex19del
-
-
Fbxl3Y-F:
+
-
-
Fbxl3:
+
gGldc:
+
+
+
aFBXL3
ab-actin
Fig. S5. Dasatinib sensitizes the antitumor effects of PD-1 blockade therapy. Related to Figure 7.
Effects of FBXL3 mutations on MHC-I surface expression. Fbxl3-deficient (gFbxl3) cells were reconstituted with mouse wild-type Fbxl3 or Fbxl3Y306F and then analyzed by flow cytometry with the indicated antibodies. Graph shows mean ± SEM, n = 3 independent samples. Data were analyzed using two-way ANOVA with GraphPad Prism 8. Lysates of the indicated cells were analyzed by immunoblots with the indicated antibodies.
